# Functional Stability of Unliganded Envelope Glycoprotein Spikes among Isolates of Human Immunodeficiency Virus Type 1 (HIV-1)

**DOI:** 10.1371/journal.pone.0021339

**Published:** 2011-06-27

**Authors:** Nitish Agrawal, Daniel P. Leaman, Eric Rowcliffe, Heather Kinkead, Raman Nohria, Junya Akagi, Katherine Bauer, Sean X. Du, Robert G. Whalen, Dennis R. Burton, Michael B. Zwick

**Affiliations:** 1 Department of Immunology and Microbial Science, The Scripps Research Institute, La Jolla, California, United States of America; 2 AltraVax, Inc., Sunnyvale, California, United States of America; 3 IAVI Neutralizing Antibody Center, The Scripps Research Institute, La Jolla, California, United States of America; 4 Ragon Institute of Massachusetts General Hospital, Massachusetts Institute of Technology, and Harvard, Boston, Massachusetts, United States of America; Institut Pasteur, France

## Abstract

The HIV-1 envelope glycoprotein (Env) spike is challenging to study at the molecular level, due in part to its genetic variability, structural heterogeneity and lability. However, the extent of lability in Env function, particularly for primary isolates across clades, has not been explored. Here, we probe stability of function for variant Envs of a range of isolates from chronic and acute infection, and from clades A, B and C, all on a constant virus backbone. Stability is elucidated in terms of the sensitivity of isolate infectivity to destabilizing conditions. A heat-gradient assay was used to determine T_90_ values, the temperature at which HIV-1 infectivity is decreased by 90% in 1 h, which ranged between ∼40 to 49°C (n = 34). For select Envs (n = 10), the half-lives of infectivity decay at 37°C were also determined and these correlated significantly with the T_90_ (p = 0.029), though two ‘outliers’ were identified. Specificity in functional Env stability was also evident. For example, Env variant HIV-1_ADA_ was found to be labile to heat, 37°C decay, and guanidinium hydrochloride but not to urea or extremes of pH, when compared to its thermostable counterpart, HIV-1_JR-CSF_. Blue native PAGE analyses revealed that Env-dependent viral inactivation preceded complete dissociation of Env trimers. The viral membrane and membrane-proximal external region (MPER) of gp41 were also shown to be important for maintaining trimer stability at physiological temperature. Overall, our results indicate that primary HIV-1 Envs can have diverse sensitivities to functional inactivation *in vitro*, including at physiological temperature, and suggest that parameters of functional Env stability may be helpful in the study and optimization of native Env mimetics and vaccines.

## Introduction

Envelope glycoprotein (Env) spikes on HIV-1 are the molecular mediators of viral entry and the sole targets on the virus of entry inhibitors and neutralizing antibody. Env is challenging for molecular studies and intervention strategies, as it is associated with high genetic diversity and significant molecular heterogeneity. Eliciting HIV-1 neutralizing antibody to primary isolates through vaccination has been particularly problematic [1], [2], [3], [4]. Neutralization of HIV-1 fails to correlate with antibody affinity to described soluble Env mimetics, but has been related to antibody recognition of native Env spikes, *i.e.* mature, membrane-associated trimers of gp120-gp41 heterodimers [4], [5], [6], [7], [8]. Still, uncertainties remain about which Env structures are functional and which are not [8], [9], [10], making it difficult to establish clear structure-function relationships. Such information is however crucial for the rational development of native Env-based mimetics, vaccines and entry inhibitor drugs.

Biosynthesis of HIV-1 Env begins with gp160 precursors, which oligomerize and become processed by a convertase (e.g. furin) and the glycosylation machinery of the host [11], [12]. Mature trimers of gp120-gp41 heterodimers then incorporate onto the membrane of infected cells and budding virions [13]. Virion-associated Env trimers engage host cell CD4 receptors [14], [15], and coreceptors (*i.e.* CCR5 or CXCR4) [16]. From the receptor contact sites on gp120, conformational changes propagate in Env to the fusion peptide of gp41, which inserts into the host cell membrane [17]. The process of viral entry is initiated when gp41 collapses into a ‘six-helix bundle’, which promotes fusion of the opposing virus and host cell membranes [17].

The HIV-1 fusion process, outlined above, arguably demands that Env trimers be labile. Indeed, a tendency for Env to dissociate is evidenced by several observations. Ionic detergent disassembles mature Env into gp120 monomers and various oligomeric forms of gp41, although under milder detergent conditions, Blue Native (BN) PAGE can be used to visualize a trimeric species of Env [9]. Spontaneous shedding of gp120 occurs with T-cell line adapted (TCLA) strains of HIV-1, which can be accelerated in the presence of soluble CD4 [18], [19], [20], [21]. CD4 has also been revealed to cause rapid inactivation of HIV-1 Env on cell-free virions [22]. Finally, heat treatment of HIV-1 at 56°C has been shown to promote gp120 shedding [10], [23].

Co-displayed with native HIV-1 Env on virions and infected cells are non-native, but highly immunogenic species of Env that display non-neutralizing gp120 and gp41 epitopes [8], [9], [24], [25]. Such species may include unprocessed gp160, misfolded Env oligomers with possible mixed disulfides or aberrant glycosylation, gp41 stumps from which gp120 has been shed, and possibly gp120-gp41 heterodimers [8], [9], [26], [27], [28], [29]. Adding to this complexity, HIV-1 exists as a diverse quasispecies in infected individuals, and multiple viruses can infect the same cell producing progeny virions that display Envs from different parents. Although non-neutralizing monoclonal antibodies (mAbs) seem unable to efficiently recognize functional Env trimers, these mAbs do typically bind to various non-functional Env molecules [8], [10]. Human mAbs have been described that can neutralize diverse primary isolates of HIV-1 (e.g. b12, VRC01, 2G12 and PG9/16 to gp120 as well as 2F5 and 4E10 to gp41), and these are important tools with which to probe Env mimetics intended for HIV-1 vaccine development [4], [10], [30], [31], [32], [33], [34], [35]. Neutralizing activity by these mAbs has been attributed to their affinity for functional Env trimers, though each cross-reacts with non-trimeric, immature or otherwise non-functional Env molecules [4], [8], [9], [32].

Recent cryo-electron microscopy models of the HIV-1 Env spike show three apical lobes corresponding to gp120 associated (non-covalently) with three gp41 molecules anchored in the membrane, in some cases leaving a solvent accessible hole about the trimer axis [36], [37], [38], [39]. At the base of the spike abutting the viral membrane is the membrane-proximal external region (MPER) of gp41 that has been suggested to be in a trimer stalk configuration [36], [38], [40] or to have a tripod structure [37], [41]. Trimer models based on the core structure of gp120 show surfaces that are either exposed to solvent, buried within trimer interfaces, or occluded by glycans [42], [43]. Mutagenesis in Env has been used to suggest that gp41 interacts with elements of the N- and C-terminal domains of gp120 [42], [44], [45], [46], [47]. In particular, the central ectodomain region of gp41 most likely interacts with C5 of gp120, as shown by engineering of a productive intermolecular disulfide link between gp41 and gp120, termed “SOS” [44], [46], [48], [49], [50].

Stability of proteins and their complexes is often determined using denaturation (unfolding) experiments, typically involving heat or treatment with chaotropic denaturants. Here, whole HIV-1 virions bearing different Envs on a common backbone were exposed to a heat gradient infectivity assay that is analogous in format to a typical neutralization assay, and the virions examined for infectivity and Env stability. In certain cases, HIV-1 was also subjected to denaturants and to prolonged incubation at 37°C. A considerable distribution from relatively thermostable to quite thermolabile Envs was revealed among (acute) primary isolates of clades A, B and C. We further showed that HIV-1 virions typically inactivated prior to complete trimer dissociation, that most highly thermolabile Envs were also functionally unstable even at physiological (37°C) temperature, as well as that an intact viral membrane and MPER of gp41 help maintain spike stability. A molecular understanding of the stability of functional Env is central to an understanding of its evolutionary fitness, and may also aid in the study and development of native Env mimetics and vaccines that are stable to specific conditions *in vitro* and *in vivo*.

## Materials and Methods

### Plasmids and antibodies

Env-deficient, molecularly cloned backbones of HIV-1, used for pseudotyped virus (PSV) production, including pSG3Δenv [51], pNL4-3.Luc.R-E- [52] and Q23Δenv [53] were obtained through the NIH AIDS Research and Reference Reagent Program (ARRRP), Division of AIDS, NIAID from J. Kappes/X. Wu, N. Landau and J. Overbaugh, respectively. The Env expression vector pSVIIIexE7pA^−^ [54], which bears *rev* and *env* under control of the natural HIV-1 long terminal repeat (LTR), was a gift from J. Sodroski (Harvard). The pSVIIIexE7pA^−^ vector has been modified in order to subclone different *env* genes as *Kpn*I-*Xho*I fragments, and these are designated pSVIII-JR-CSF, pSVIII-ADA, pSVIII-JR-FL, pSVIII-JR2, etc. [55]. HIV-1 gp160_JR2_ is identical to that of JR-FL but has three substitutions in the membrane-proximal external region (MPER) of gp41 (*i.e.*, S668N, T676S, and K677N), as described previously [56]. Acute phase HIV-1 Env panels of clades B and C were obtained from the ARRRP (J. Mascola, D. Montefiori, B. Hahn, E. Hunter and L. Morris) [51], [57], [58], and the clade A panel was a kind gift from J. Overbaugh [59]. Env from SF162 is expressed in pCAGGS-SF162, a gift from J. Binley (Torrey Pines Institute for Molecular Studies), as is that of SIVmac239 that has a stop codon at position 718 which truncates the cytoplasmic tail [60]. pVSV-G [61] was a gift from T. Friedmann (UCSD).

Antibodies used in this study were obtained from the following sources (epitope specificities in parentheses): IgGs 2G12 (gp120 high-mannose glycans) [31], 4E10 and 2F5 (MPER) [62] were gifts from H. Katinger (Polymun); IgGs b12 (CD4 binding site; CD4bs) [30], b6 (CD4bs) [8], and Z13e1 (MPER) [63] were produced in house; IgG 7B2 (gp41, principal immunodominant domain) [9] was a gift from J. Robinson (Tulane); IgG F425 B4e8 (V3) [64] was a gift from L. Cavacini (Harvard Medical School). Four-domain soluble human CD4 (sCD4, contributed by Progenics Pharmaceuticals) was obtained from the NIH ARRRP.

### Construction of pLAI-BS vector

HIV-1 molecular clone (MC) pLAI.2, contributed by K. Peden, was obtained from the NIH ARRRP [65]. For directional subcloning of Env (gp140) ectodomains into a common HIV-1 backbone, pLAI.2 was modified. The sequence of the modified vector, pLAI-BS, is available upon request. Thus, an existing *Bgl*I site in pLAI.2 (nucleotide 10702 in the *bla* gene) was knocked out using Quikchange mutagenesis (Stratagene). Meanwhile, the *Nco*I-*Xho*I fragment of pLAI.2 was subcloned into a shuttle vector, pET-20b(+) (Novagen), and an existing *BamH*I site (nucleotide 8569) was mutated from (GGATCC) to (GGATTC) using Quikchange, and unique *BamH*I and *Sfi*I/*Bgl*I sites were silently introduced into the leader sequence (position 6372) and transmembrane domain of *env* (nucleotide 6712), respectively. A second *Bam*HI site was similarly introduced into *env* (nucleotide 8055) and a 1683 bp *BamH*I fragment was excised from the *Nco*I-*Xho*I stuffer fragment. The resulting *Nco*I-*Xho*I stuffer fragment was subcloned back into pLAI-2 (now *Bgl*I- deficient, as described above) to render pLAI-BS, a destination vector incapable of producing infectious virus until a *Bam*HI-*Bgl*I fragment, corresponding roughly to gp140, is introduced. Several heterologous gp140-fragments from R5-tropic isolates (SF162, ADA, JR-FL, JR2 and JR-CSF) were introduced into pLAI-BS using PCR amplification and subcloning *via* the unique *BamH*I and *Sfi*I/*Bgl*I sites. Resulting chimeric LAI MCs were used to generate virus stocks, as described below. All plasmids not obtained from the ARRRP were sequence-verified in *env*.

### Cells and viruses

TZM-bl cells were obtained from the NIH ARRRP, as contributed by J. Kappes and X. Wu [51]. HEK293T cells were obtained from the American Type Culture Collection. Both cell lines were maintained in Dulbecco's modified Eagle's medium (DMEM; Invitrogen) containing 10% heat-inactivated fetal bovine serum, 20 mM glutamine, 100 U/ml penicillin and 100 µg/ml streptomycin. MT-2/CCR5ΔCT cells are MT-2 (CXCR4^+^) cells that have been transfected and selected to stably express high levels of cytoplasmic tail-truncated CCR5 receptors (a gift from D. Mosier, TSRI) [66]. MT-2/CCR5ΔCT cells were cultured in RPMI 1640 supplemented with 10% heat-inactivated fetal bovine serum, 20 mM glutamine, 100 U/ml penicillin and 100 µg/ml streptomycin (Invitrogen). Peripheral Blood Mononuclear Cells (PBMC) were purified from a pool of at least 3 donors from the Scripps Normal Blood Donor program using a Ficoll-Hypaque method according to the manufacturer's instructions (Sigma). Mammalian cell cultures were incubated at 37°C in a humidified 5% CO_2_ environment.

HIV-1 PSVs were produced by co-transfection of HEK293T cells using an Env complementation plasmid and either pNL4-3-Luc, Q23Δenv, or pSG3Δenv at a 1∶2 ratio. Replication competent HIV-1 was produced by transfection using the appropriate pLAI-Env chimeric MC. In general, cells were transfected at 70–95% confluency using polyethanolimine (PEI; 25 kDa, Sigma-Aldrich) diluted in serum free Opti-MEM (Invitrogen) at a DNA∶PEI ratio of 1∶3.5 [67]. Virus-containing supernatants were collected at 48 and/or 72 h post-transfection, cleared of cellular debris by centrifugation at 3,000× *g* for 15 min, sterile filtered (0.2 µm) and either used immediately, or frozen no more than once at −80°C. Virus-containing supernatants were concentrated, as necessary, by centrifugation at 20,000× *g* for 45 min at 4°C, followed by resuspension of the viral pellet in an appropriate volume of PBS.

HIV-1 MCs were amplified and cultured in MT-2/CCR5ΔCT cells or PBMCs. Cells were infected at an m.o.i. of 0.01. Virus containing supernatants were harvested when maximum titer was reached, as determined using the TZM-bl cell infectivity assay, usually 7–9 days post infection.

### HIV-1 infectivity assay (TZM-bl)

HIV-1 infectivity was determined as described previously [51]. Briefly, TZM-bl cells were seeded in 96-well plates at 10^4^ cells per well in 100 µl complete DMEM and incubated for 24 h at 37°C. Virus samples were added to cells in a total volume per well of 200 µl DMEM. Cells were harvested 48 h post-infection, and HIV-1 LTR-induced luciferase activity in the cells was determined using the Luciferase Assay System (Promega). Results are reported in relative luminescence units (RLU) as measured on an Orion microplate luminometer (Berthold Instruments).

### ELISAs

#### (i) p24(Gag) ELISA

Microtiter wells were coated overnight at 4°C with 50 µl of 5 µg/ml sheep anti-p24 (Aalto) and blocked using 4% non-fat dry milk (NFDM) in PBS. Virions were lysed by adding 1% Empigen (final concentration) in PBS and 50 µl were transferred to anti-p24-coated wells. After incubating for 2 h at 37°C, p24 was probed using alkaline phosphatase conjugated sheep-anti-p24 (Aalto), and the assay developed using the AMPAK amplification kit (Argene), according to the manufacturer's directions.

#### (ii) gp120 ELISA

gp120 ELISAs were performed as p24 ELISAs with the following exceptions: microtiter wells were coated with *Galanthus nivalis* lectin (GNL; 5 µg/ml in PBS), and probed using an anti-gp120 monoclonal antibody (mAb) cocktail (b12 and B4e8; 1 µg/ml each) followed by detection using an HRP-conjugated anti-human Fc (Jackson) secondary reagent. The colorimetric signal was produced using the TMB substrate (Pierce) and absorbance at 450 nm was measured using a microplate reader (Molecular Devices).

### HIV-1 reverse transcriptase activity assay

The HIV-1 reverse transcriptase activity assay was performed as described by the manufacturer (Colorimetric Reverse Transcriptase Assay, Roche).

### Temperature gradient infectivity assay for HIV-1

To determine sensitivity of HIV-1 infectivity to heat treatment, a gradient PCR block (Mastercycler Gradient, Eppendorf) was used. MC and PSV virion samples were incubated in parallel on a thermal gradient (12 temperatures) from 37 to 57°C for 1 h. Temperature accuracy of the block was verified using a K element probe (Ebro) in all wells. Virus-containing samples were loaded onto a 96-well PCR plate (100 µl per well). Following heat treatment, samples were allowed to cool to RT prior to determination of HIV-1 infectivity using TZM-bl cells. Samples that produced RLUs below 150,000 or above 1,500,000 following a 1 h treatment at 37°C were considered outside of the linear range of the assay and were excluded. For determination of T_90_, RLU data were background-subtracted using RLU counts generated from uninfected cells (typically ∼3,000 RLU) and normalized according to the 37°C data point. The assay is not considered sensitive at or below 0.1% maximal infectivity, so all values at or below this level of infectivity were designated 0.1% infectivity for plotting purposes. This adjustment does not affect the T_90_ value or any conclusions derived from the data. Replicate data were curve fitted using either a cubic spline or log dose response equation with similar outcomes, and the temperature corresponding to 90% reduction of infectivity relative to treatment at 37°C was determined using Prism version 5.0 software (Graphpad, CA).

### HIV-1 infectivity half-life (t_1/2_) at 37°C

HIV-1 (PSV) in cleared cell culture supernatants were aliquoted undiluted, and diluted 1∶5 in DMEM, into 1.5 ml polypropylene tubes corresponding to 4 different time points: t_0_, t_5 h_, t_20 h_, t_44 h_. Following incubation, samples were frozen at −80°C, then synchronously thawed and HIV-1 infectivity was determined using TZM-bl cells as described above. Replicate infectivity data were background subtracted (average 2,000 RLU), analyzed using a non-linear curve fit, one phase exponential decay equation (plateau constraint = 0), and the time corresponding to 50% reduction of infectivity relative to t_0_ was determined using Prism version 5.0 software (Graphpad, CA). Maximal HIV-1 infectivities were kept within a linear dynamic range between 100,000–1,500,000 RLU. Experiments were performed in at least triplicate and produced a goodness of fit, r^2^≥0.90.

### Treatment of HIV-1 with guanidinium hydrochloride (GuHCl), urea and extreme pH

Virus samples were treated using a range of concentrations of freshly prepared GuHCl (G9284, Sigma) or urea (U4883, Sigma) or pH buffer (25 mM citric acid and 25 mM sodium citrate for pH 3–6.5, and 25 mM ethanolamine HCl for pH 6.5–10). Microcentrifuge tubes containing virus and denaturant or pH buffer were immediately centrifuged for 45 min at 20,000× *g* at 4°C to pellet the virus. The supernatant was carefully removed and the virus pellet washed twice with sterile PBS to remove residual denaturant or pH buffer. The final pellet was resuspended in 100 µl DMEM and the virus titered using the TZM-bl infectivity assay.

### Blue Native (BN) PAGE

Virus samples were concentrated by 100-fold, treated with 1% DDM (n-Dodecyl-ß-maltoside; Invitrogen) for 20 min on ice, then subjected to electrophoresis on 4–16% NativePAGE Bis-Tris gels, according to the manufacturer's instructions (Invitrogen). Samples were separated at RT using 120 V with dark blue cathode buffer until the dye front migrates 1/3rd of the way down the gel. The dark blue buffer was immediately replaced with light blue cathode buffer and the electrophoresis resumed for the remainder the gel. Proteins were then transferred to a PVDF membrane under wet conditions using transfer buffer containing 10% methanol for 1 h at 20 V on ice. Membranes were fixed for 20 min with 10% acetic acid and 25% methanol in water. Excess Coomassie dye was removed by incubation in methanol twice for 10 min. Membranes were blocked in blocking buffer (4% NFDM, 0.1% Triton X-100 in PBS) for 30 min at RT and probed overnight at 4°C using primary antibodies to gp120 (b12, 2G12 and b4e8 each at 2 µg/ml) or to gp41 (2F5, 4E10 and Z13e1 each at 1 µg/ml) diluted in blocking buffer. The membranes were vigorously washed four times for 10 min each in wash buffer (0.05% Tween 20 in PBS) and incubated for 30 min at RT with a horseradish peroxidase-conjugated goat anti-human Fc antibody (diluted 1∶3,000 in blocking buffer). The membranes were washed as above, rinsed in water, and HRP substrate (Super Signal West Pico Chemiluminescence, Pierce) was added. Membranes were exposed to film (Genesee Scientific) that was analyzed using a GS-800 Densitometer (Bio-Rad).

### SDS-PAGE

Concentrated virus samples were incubated in Laemmli Buffer (Bio-Rad) containing 50 mM dithiothreitol (DTT) for 5 min at 100°C prior to loading on 4–15% Tris-HCl (Bio-Rad ReadyGel). Protein samples were separated in the gel at RT using running buffer (25 mM Tris, 192 mM Glycine, 0.1% SDS) at 120 V for 1.5 h, then transferred to PVDF membrane and processed using a similar procedure as for BN-PAGE.

### Effect of detergent-solubilization and β-cyclodextrin on Env by BN-PAGE

Virus samples of were concentrated 100-fold, and were treated with 1% DDM (n-Dodecyl-ß-maltoside; Invitrogen) or 70 mM 2-hydroxypropyl-β-cyclodextrin (Sigma) prior to incubation at 4 different time points: t_0_, t_4 h_, t_8 h_, t_24 h_, t_96 h_. Following incubation, samples were frozen at −80°C, then synchronously thawed and subjected to BN-PAGE on 4–16% NativePAGE Bis-Tris gels (Invitrogen), as described above.

### In-solution virus capture assay

The capture assay was performed as previously described [10]. Briefly, microtiter wells were coated overnight at 4°C with polyclonal anti-Fc (Jackson; 5 µg/ml in 50 µl of PBS). Wells were blocked with 4% non-fat dry milk in PBS for 1 h at 37°C. Capture mAbs were added to 50 µl of virus and incubated for 2 h at 37°C in microcentrifuge tubes. Virions were pelleted at 20,000× *g* for 45 min at 4°C in a microcentrifuge, resuspended in an equal volume of PBS, and 50 µl was added to blocked microtiter wells previously coated with the immobilized anti-Fc. Following a 1 h incubation at 37°C, wells were washed 6 times with PBS and virus equivalents quantified by p24 ELISA (see above).

## Results

### Determination of HIV-1 infectivity following heat treatment

**Figure 1:**
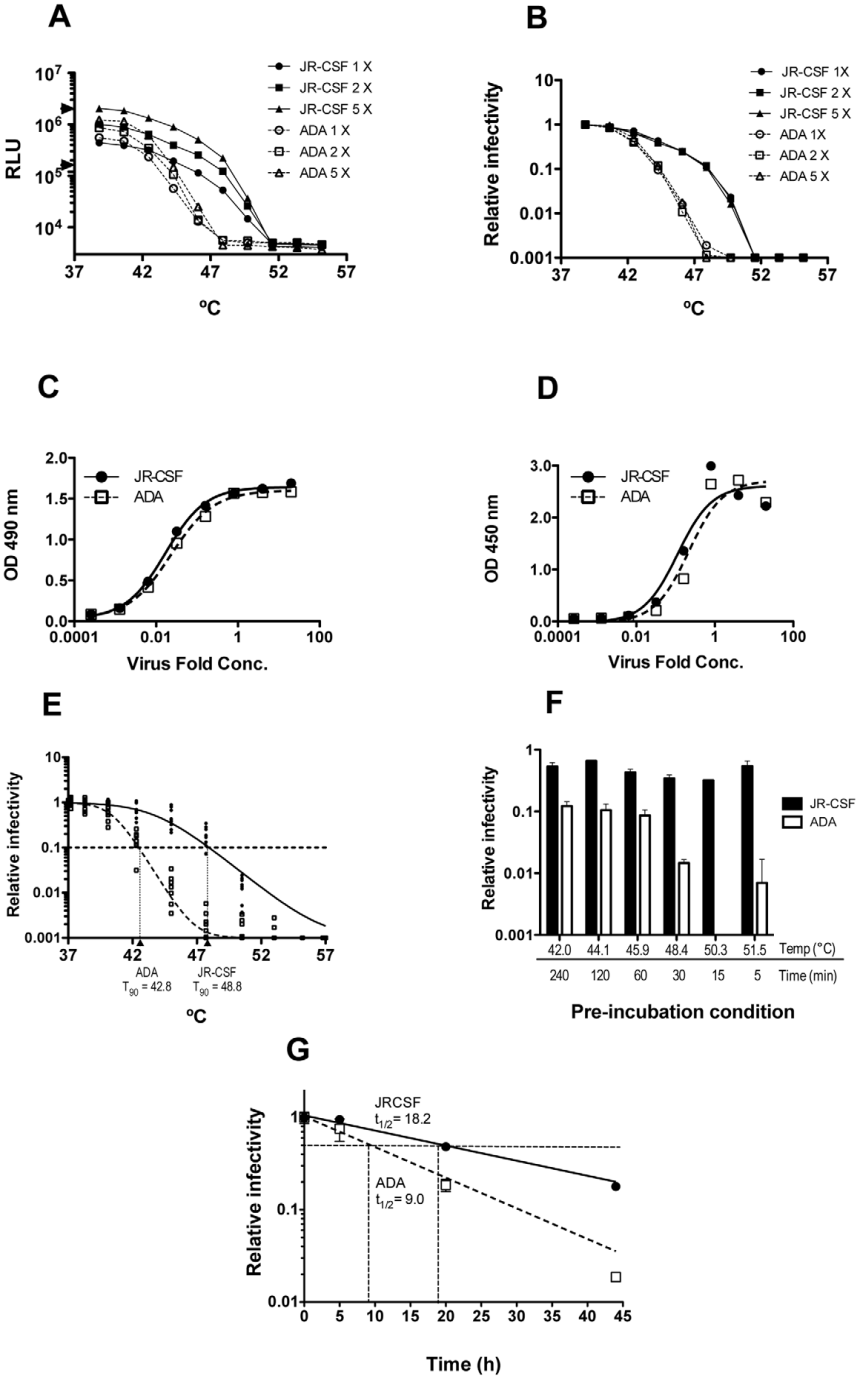
Sensitivity of HIV-1 to heat is Env-dependent. HIV-1 PSVs on a pSG3Δenv backbone and bearing Env from strains ADA or JR-CSF, were concentrated (20×) in PBS and incubated for 1 h at 1, 2 or 5 times their original concentration at various temperatures for 1 h prior to determination of infectivity using TZM-bl cells. (**A**) Absolute infectivity following heat treatment of HIV-1 PSVs at different virus input levels over a >10-fold range (1×10^5^–2×10^6^ RLU/0.1 ml, demarcated by triangles). (**B**) Data from panel A normalized for input infectivity, indicating T_90_ value is independent of virus input. HIV-1 PSVs used in panel A were examined for relative levels of p24 (**C**), and gp120 (**D**), using p24 or gp120 ELISA, respectively. (**E**) Reproducibility of T_90_ determination using independent stocks of HIV-1 PSVs (JR-CSF and ADA). (**F**) Relative infectivities of HIV-1 PSVs (JR-CSF and ADA) following several different pre-incubation temperatures and times, as determined using the TZM-bl assay. (**G**) Relative infectivity over time at physiological temperature (37°C) of HIV-1 PSVs (JR-CSF and ADA). Half-life (t_1/2_) of infectivity was calculated as described in the Materials and Methods and is indicated near the point of half-maximal infectivity for each curve.

To determine the influence of Env stability on HIV-1 infectivity, we began by subjecting virions that differed only in Env to heat and then determined infectivity using TZM-bl reporter cells [51]. As the HIV-1 backbone remains constant and only Env is varied, differences in sensitivity to heat of the chimeric virions will most likely relate to thermostability of Env. HIV-1 pseudotyped virions (PSVs) were produced by transfection of 293T cells using the pSG3Δenv vector and an Env-complementation vector [51]. Pilot experiments using microcentrifuge tubes in a heat block revealed that HIV-1_JR-CSF_ was more stable to heat treatment than HIV-1_ADA_ (data not shown). To assign a quantitative parameter to this observation in a rapid screen format, we employed a gradient PCR block with temperatures evenly distributed from 37–57°C. A heat inactivation curve was generated and a T_90_ was designated as the temperature at which HIV-1 infectivity decreases by 90% in 1 h. Following multiple experiments, we consistently found that HIV-1_JR-CSF_ and HIV-1_ADA_ had T_90_ values averaging around 48.8°C and 42.8°C, respectively, whether virions were normalized for p24, Env or infectivity (**Fig. 1A–E**, and data not shown). We also found that HIV-1_ADA_ was more thermosensitive than HIV-1_JR-CSF_ using various fixed incubation times and temperatures, as well as prone to more rapid decay at 37°C (**Fig. 1F and 1G**). Thus, we found the thermal gradient infectivity assay to be a rapid means of comparing thermostabilities (T_90_ values) of HIV-1 Env variants on a constant backbone.

### Thermostability of HIV-1 Env ectodomain variants with different backbones

HIV-1 backbone components, such as the matrix protein (p17) on the inner leaflet of the viral membrane that interacts with the cytoplasmic tail of gp41 [13], could conceivably influence the T_90_ values in our assay. Hence, we compared T_90_ values of several HIV-1 PSVs produced using either pSG3Δenv, or a heterosubtypic HIV-1 backbone, Q23 (Clade A; [68]), with that of replication-competent molecular clones (MCs), the latter of which are chimeras of HIV-1_LAI_ [65] engineered with heterologous Env ectodomains. (Amino acid sequence identity for p17 between pLAI and pSG3 is 89.4% and between Q23 and the clade B backbones is 78%.) We found that reverse transcriptase (RT) thermostabilities of four different backbones (*i.e.* pSG3ΔEnv, Q23, pLAI-2 and pNL4-3Luc) following a 1 h incubation at various elevated temperatures were indistinguishable (data not shown). Ninety-percent reduction in RT activity occurred at a pre-incubation temperature of ∼52°C, which is slightly more thermostable than the HIV-1 variant Envs tested (T_90_s<49°C; **Fig. 1**, **Table 1** and data not shown). Overall, viruses produced using the Q23 backbone were similar in thermostability to their pSG3 or pLAI counterparts, although minor differences in T_90_ values (usually 1–2°C or less) were occasionally observed (**Table 1**). Importantly, LAI-Env MC chimeras were also found to have identical stabilities as their parental Env counterparts in the PSVs (**Table 1**), even though the MCs contain sequences in Env that originate from the LAI strain, including the Env leader sequence and gp41 transmembrane and cytoplasmic tail (CT) domains. Truncation of the gp41 CT, which eliminates Gag-Env interactions altogether [69], had a relatively minor effect on the T_90_ of HIV-1_JR-FL_ (46.1 c.f. 47.4°C; data not shown). Collectively, the above results show that the observed differences in T_90_ values, when Env is varied on a constant HIV-1 backbone, depend mainly on sequence differences in the Env ectodomain, irrespective of whether PSVs or MCs were used.

**Table 1:** Sensitivity of HIV-1 infectivity to heat (T_90_) is linked to Env.

Env	T_90_ (°C) (± SEM)^1^ of HIV-1 using different backbones			
	Pseudotyped virus (PSV)^2^		Molecular clone (MC)^3^	
	pSG3Δenv	Q23Δenv	293T	MT-2/CCR5ΔCT
JR-CSF	48.8±0.5	47.7±0.6	48.2±0.2	48.5±0.5
JR-FL	47.7±0.5	47.4±0.5	46.7±0.5	45.7±0.6
SF162	44.4±0.4	46.7±0.7	44.2±0.5	45.3±1.4
ADA	42.8±0.3	43.6±0.3	42.0±0.2	43.7±1.8
LAI	44.9±0.4	44.7±0.2	44.3±0.2	*nd* 4
92UG037.8	45.6±0.5	47.6±0.4	*nd*	*nd*
Q23.17	42.5±0.5	42.9±0.7	*nd*	*nd*
SIVmac239	41.1±1.3	42.2±0.3	*nd*	*nd*
VSV-G	43.4±0.2	43.0±0.2	*nd*	*nd*

1 T_90_, the temperature at which HIV-1 infectivity decreases by 90% in 1 h, and the standard error of the mean (SEM) of at least three independent experiments.

2 HIV-1 PSVs, produced by transfection of 293T cells using *env* expression plasmids pSVIIIexe7 (JR-CSF, JR-FL, ADA), pCAGGS (SF162, SIVmac239), pcDNA3.1 (LAI), or pVSV-G (VSV-G) in combination with the backbone vectors pSG3Δenv (clade B) or Q23Δenv (clade A).

3 HIV-1 MCs are LAI-chimeras (see Materials and Methods) and fully replication competent. Virions were produced by transfection of 293T cells, or through passage in MT-2/CCR5ΔCT cells, as indicated.

4 *nd*, not determined, or material unavailable.

We also propagated chimeric HIV-1 MCs in a cell line, MT-2/CCR5ΔCT, that has been engineered to stably express a cytoplasmic tail-truncated CCR5 [66]. The T_90_ values of MT-2/CCR5ΔCT-passaged viruses followed the same trend of most to least stable isolates, *i.e.* JR-CSF≧JR-FL>SF162>ADA, indicating that Env genotype again dictated the T_90_, whether 293T or MT-2/CCR5ΔCT were used as producer cells (**Table 1**). Notably, HIV-1 pseudotyped using Env from SIVmac239 or vesicular stomatitis virus (VSV-G) were found to have relatively low T_90_ values of 42°C and 43.4°C, respectively. Thus, HIV-1 variant Envs were more thermostable than that of the other two enveloped viruses, at least on an HIV-1 backbone.

### Sensitivity of HIV-1 to denaturants and pH

**Figure 2:**
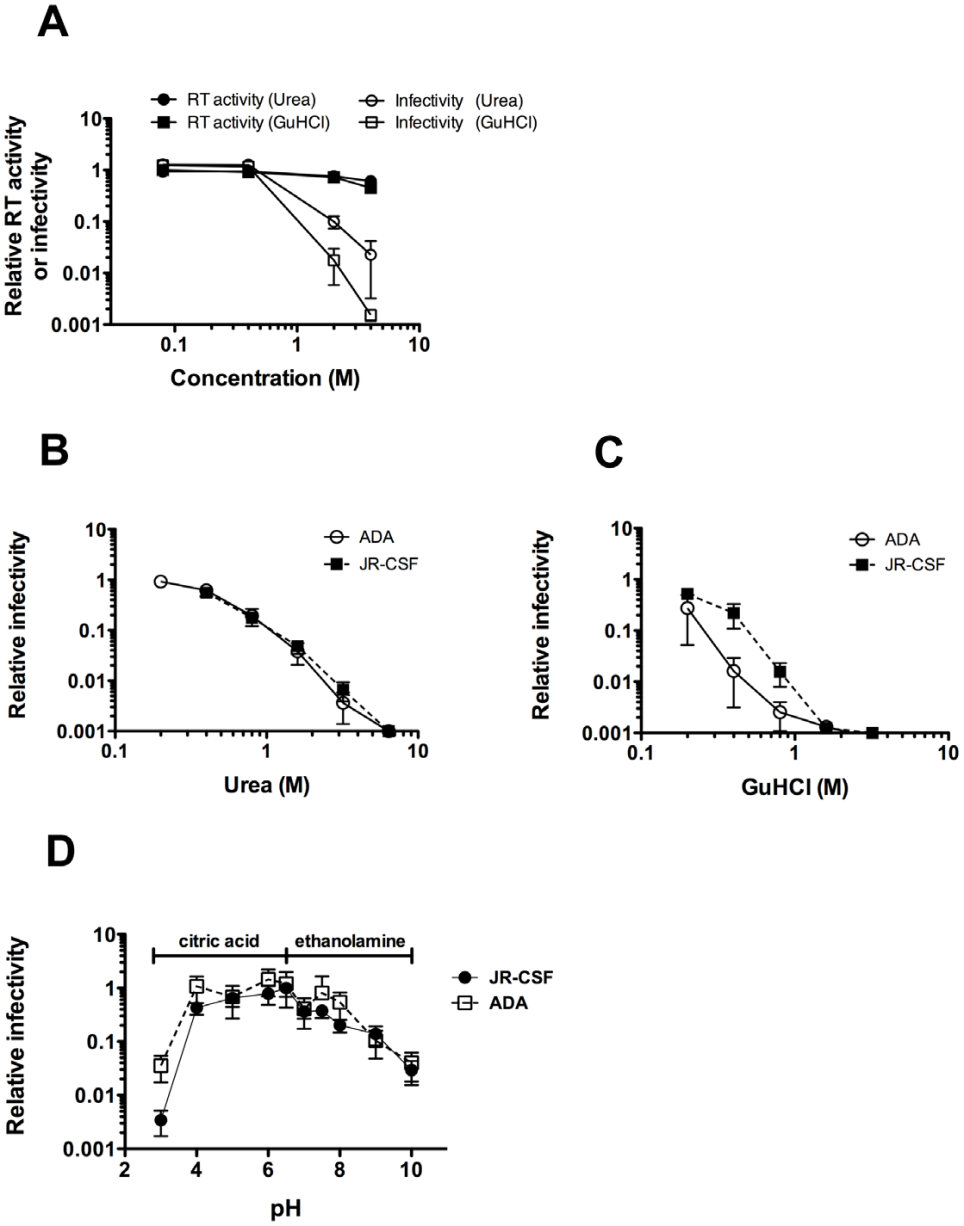
Sensitivity of HIV-1 to urea and GuHCl. (**A**) Treatment of HIV-1 with denaturant abrogates virion infectivity under conditions in which virion-associated RT remains active. Culture supernatants containing infectious HIV-1_LAI-JR-CSF_ (MC) virions, passaged once in MT-2/CCR5ΔCT cells, were treated using different concentrations of urea and GuHCl. Following extensive washing to remove denaturant, samples were assayed for apparent RT activity (closed symbols) as well as for infectivity on TZM-bl cells (open symbols). Data plotted are normalized to untreated samples. (**B** and **C**) HIV-1 (PSVs) produced in 293T cells using backbone plasmid pSG3Δenv, and Env plasmids pSVIII-JR-CSF (solid line) and pSVIII-ADA (dotted line) were incubated with indicated concentrations of (**B**) urea, or (**C**) GuHCl. Prior to infectivity determination using TZM-bl cells, virions were pelleted and washed with PBS to remove residual denaturant. Results are an average of duplicate samples, and representative of at least two independent experiments. (**D**) Sensitivity of HIV-1 to pH. HIV-1 PSVs, prepared and treated as in **Fig. 3**, except that citric acid (pH 2–6.5) or ethanolamine (pH 7–10) buffers were used. Results are an average of duplicate samples, and representative of three independent experiments.

In assaying protein stability, the chaotropic denaturants, urea and guanidinium hydrochloride (GuHCl), are commonly used. Whereas heat will indiscriminately penetrate into the interior of Env and other components of HIV-1, such as the RNA polymerase, denaturants are expected to interact mainly with solvent-exposed Env. We also wished to determine how pH might affect functional Env stability. To avoid toxicity to target cells, we treated HIV-1 with denaturants or pH buffers, and then pelleted virions to wash away residual supernatant, prior to infecting cells. As expected, both urea and GuHCl diminished infectivity of PSVs in a concentration-dependent manner, relative to treatment with PBS alone (**Fig. 2A**). At concentrations of urea and GuHCl that abrogated HIV-1 infectivity, RT activity was only diminished by 50% or less (**Fig. 2A**), suggesting that the denaturants did not penetrate and disrupt virion cores. Interestingly, the thermolabile HIV-1_ADA_ was found to be equally as sensitive to urea as the more thermostable HIV-1_JR-CSF_ (**Fig. 2B**), whereas HIV-1_ADA_ was more sensitive than HIV-1_JR-CSF_ to GuHCl (**Fig. 2C**). Moreover, little to no difference was observed between HIV-1_ADA_ and HIV-1_JR-CSF_ in sensitivity to pH across a large pH range (pH 4–10) (**Fig. 2D**). Thus, with HIV-1_ADA_, heat, 37°C incubation and GuHCl treatment were hyper-destabilizing, but urea and pH were not, indicating that some specificity exists in the effect of destabilizing conditions with particular Envs.

### Excess unprocessed gp160 in virion preparations has no effect on HIV-1 thermostability

**Figure 3:**
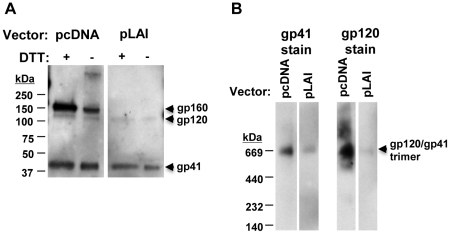
Presence of an excess of unprocessed gp160 in PSV but not MC HIV-1 virion preparations. (**A**) SDS-PAGE of HIV-1_LAI-JR-CSF_ PSVs (pcDNA) showing excess of uncleaved gp160, and HIV-1_LAI-JR-CSF_ MC (pLAI; sequence matched in Env) showing only the much fainter gp120 band, as well as gp41. Virus loaded was normalized by p24 ELISA. (**B**) BN-PAGE of samples in panel A, showing mostly oligomeric Env (PSVs and MCs), with HIV-1_LAI-JR-CSF_ PSVs showing greater heterogeneity in staining with the gp120 mAb cocktail, and with MCs showing much less abundant, but mainly trimeric Env. Input virus was normalized as in **A**.

We previously found that excess, unprocessed gp160 can associate with virions and wondered whether this association may have an effect on HIV-1 sensitivity to heat [10]. Using ELISA, we measured levels of virion-associated Env and Gag (p24) for ADA and JR-CSF and found that each had similar levels of both proteins (**Fig. 1C and D**; data not shown). However, HIV-1 PSV preparations, produced using pcDNA and pSVIII Env-complementation vectors bearing promoters from CMV and HIV-1 long terminal repeat (LTR), respectively, contained 4–5-fold more total Env, as compared to Env-matched MCs (**Fig. 3**), consistent with our findings in a prior study [10]. SDS-PAGE revealed that excess Env associated with the PSVs was mostly unprocessed gp160, a portion of which was oligomeric and sensitive to DTT treatment as reported previously (**Fig. 3A**) [26]. In contrast, pLAI-chimeric MCs showed lower Env levels overall but efficient processing of gp160 (**Fig. 3A**). Importantly, PSV and MC virions were found to have indistinguishable T_90_ values (**Table 1**). We also tested and found that pLAI-chimeric (MC) HIV-1_ADA_ has a similar infectivity half-life (t_1/2_ = 9.2±1.9 h) as the HIV-1_ADA_ PSV (t_1/2_ = 9.0±0.4 h) (**Fig. 1G**, data not shown). Hence, the presence or absence of excessive uncleaved gp160 in HIV-1 preparations has no apparent effect on functional thermostability of fusion-competent Env.

### Visualization of virion-associated Env trimer dissociation using BN-PAGE

**Figure 4:**
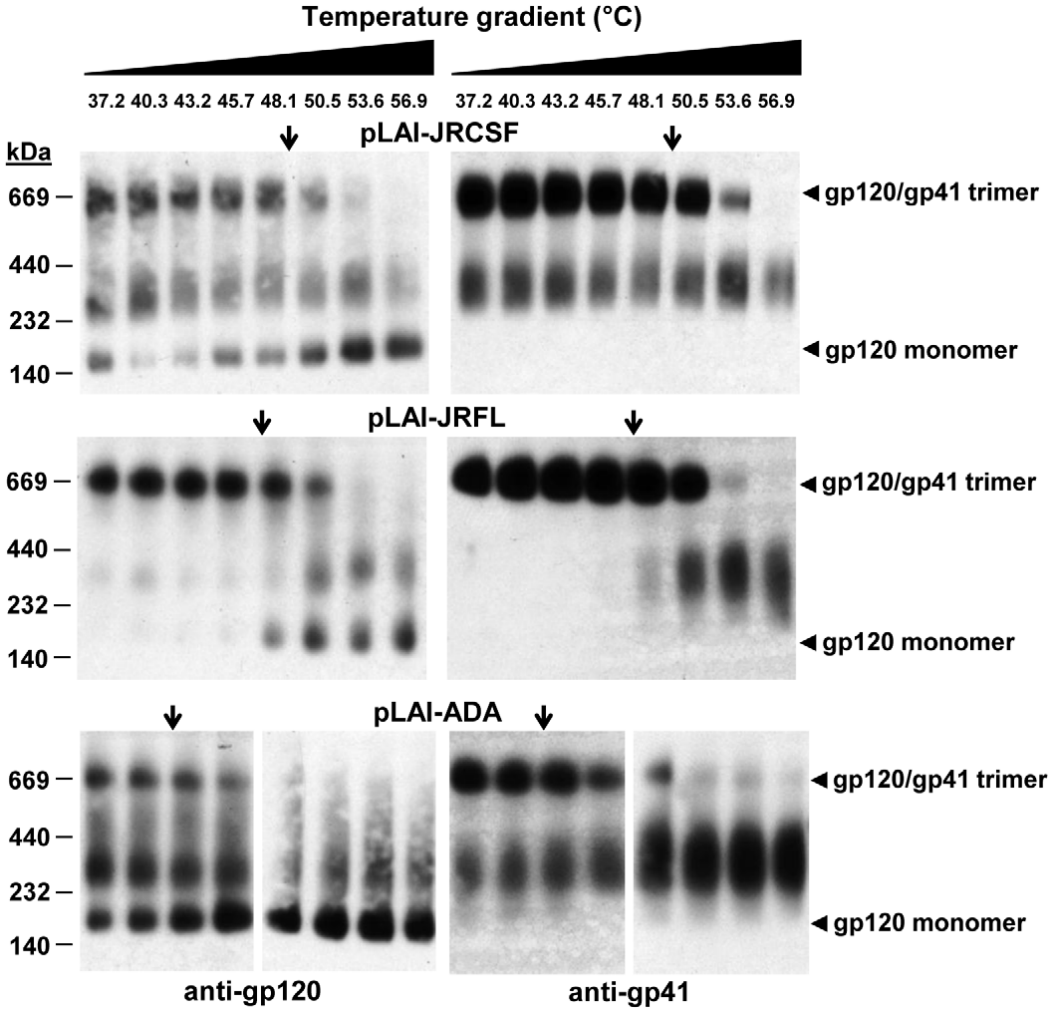
Thermally induced dissociation of HIV-1 Env trimers visualized using BN-PAGE. HIV-1 LAI-chimeric MCs bearing Envs of JR-CSF (top), JR-FL (middle) and ADA (bottom) were treated for 1 h at temperatures ranging from 37°C to 57°C, and then subjected to BN-PAGE and Western blot analysis. Blotted membranes were probed using mAb cocktails to gp120 (IgGs b12, 2G12 and B4e8) or to gp41 (IgGs 2F5, 4E10 and Z13e1). Positions of molecular weight standards are indicated (left), as are positions of monomeric gp120 and native gp120/gp41 trimers (right). The down arrow (↓) on each panel indicates the T_90_ of the cognate virus, as reported in **Table 1**.

We wished to determine whether a relationship exists between the T_90_ thermostability value and the extent of heat-induced dissociation of HIV-1-associated Env trimers. To this end, we employed BN-PAGE analysis, which can be used to separate oligomeric forms of HIV-1 Env that are liberated from virions using mild detergent [9], [70], [71]. We calibrated our BN-PAGE using gel mobility shift assays to verify that neutralizing mAbs but not non-neutralizing ones could shift the band corresponding to trimeric Env, as has been reported previously (data not shown) [9]. We found that BN-PAGE using HIV-1 PSV preparations were frequently difficult to interpret due to the presence of excess uncleaved gp160, a significant proportion of which is in an oligomeric state prior to heat treatment (**Fig. 3A and B**), and which is also relatively heat resistant [10]. We focused instead on the LAI-chimeric MCs for BN-PAGE analyses, as these typically produced >90% cleaved Env under similar conditions. When these virions were heated and separated by BN-PAGE, trimer dissociation could be visualized on blots stained using mAb cocktails against either gp120 or gp41. Notably, bands corresponding to mature Env trimers (JR-FL, JR-CSF and ADA) were found to disappear gradually at temperatures somewhat above the T_90_ (**Fig. 4**). Moreover, the rank order of trimer thermostability observed using BN-PAGE matched that of the T_90_ values (*i.e.* JR-CSF>JR-FL/JR2>ADA). The results indicate that the HIV-1 isolates inactivate in a manner dependent on Env stability and that complete dissociation of all Env spikes is not required. With HIV-1_JR-FL_ in particular, a band corresponding to monomeric gp120 is shown to accumulate over the same temperature transition in which the trimer band decays (**Fig. 4**). Monomeric gp120 also accumulated as a result of heating HIV-1_JR-CSF_ and HIV-1_ADA_, but these strains also exhibited more Env heterogeneity and spontaneously shed gp120, presumably in part as a result of BN-PAGE sample preparation, making the accumulation of monomeric gp120 more difficult to monitor with these two isolates.

BN-PAGE analysis further revealed that untreated HIV-1_JR-FL_ displays mainly trimeric Env, whereas HIV-1_JR-CSF_ and HIV-1_ADA_ produce bands corresponding to monomeric gp120 and a form of Env that migrates at a mol. wt. of ∼200 kDa (**Fig. 4**). The latter band stains with both anti-gp120 and anti-gp41 mAbs (**Fig. 4**), and its gel mobility shifts using non-neutralizing antibody (*e.g.* IgG b6 [8], [9], data not shown), so this band could be uncleaved gp160, or processed gp120-gp41 monomers or dimers, as previously described [9], [10], [28], [72]. These observed differences in heterogeneity between HIV-1 Envs were reproducible using fresh preparations of virus. Surprisingly, thermostability (T_90_) of native Env trimers did not necessarily predict the level of heterogeneity of virion-associated trimers, as both stable and labile Envs were found to be relatively heterogeneous (*i.e.* JR-CSF and ADA, respectively) whereas HIV-1_JR-FL_ is of intermediate thermostability but produces relatively homogeneous trimers.

**Figure 5:**
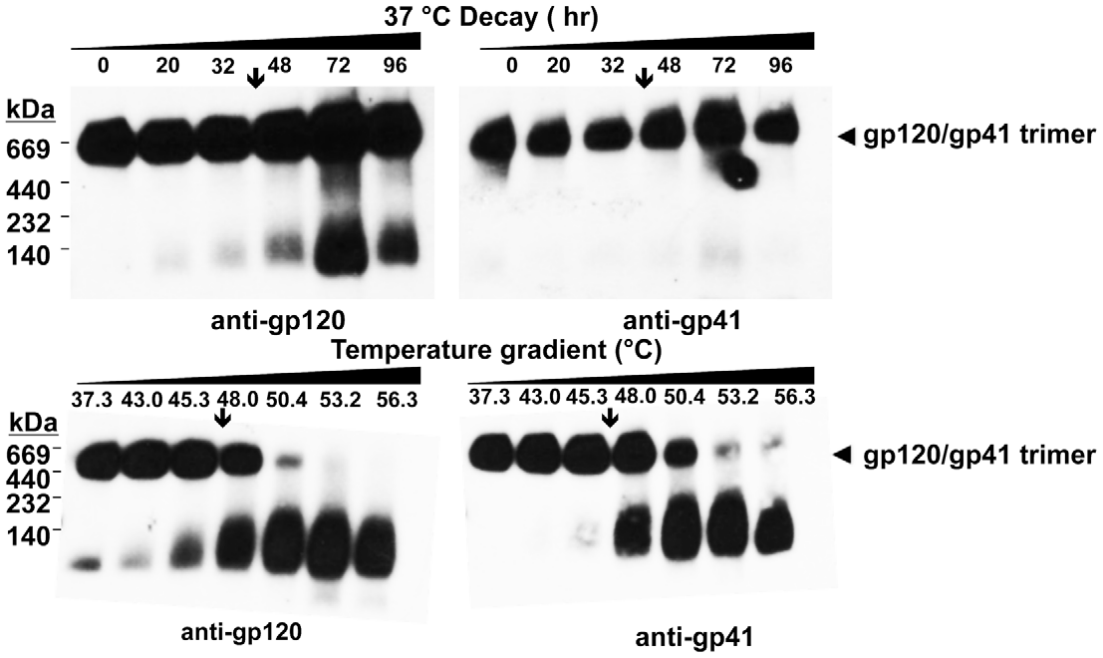
Time course incubation of HIV-1 Env trimers at physiological temperature (37°C) visualized using BN-PAGE. (**A**) HIV-1_LAI-JR2_ MC, produced in 293T cells, was incubated for various time intervals up to 96 h at 37°C and aliquots removed for BN-PAGE and Western blot analysis, as in **Fig. 4**. Down arrows indicate the time interval in which infectivity of the cognate virus decreases by ≥90%. The band smearing in the 72 h lane is an experimental artifact of sample loading. (**B**) The same virus sample as in Panel A was subjected to indicated temperatures for 1 h and analyzed on BN-PAGE, as in **Fig. 4**, except that the electrophoresis run time was shorter causing less separation between different bands.

Similar to its close homolog MC HIV-1_JR-FL_ [63], HIV-1_JR2_ was found to produce relatively homogeneous trimeric Env by BN-PAGE analyses (data not shown), and was examined for Env trimer dissociation, this time at physiological temperature (37°C). Incubation of HIV-1_JR2_ at 37°C resulted in modest trimer dissociation that accumulated over several days. Trimer dissociation was incomplete at day 4 (96 h), even though at this point infectivity had decayed to <1% the original level (**Fig. 5A**; data not shown). When the same sample of HIV-1_JR2_ was subjected to the heat gradient, extensive Env trimer dissociation was observed at a temperature just above the T_90_ (**Fig. 5B**), similar to that observed with the other Env trimers (**Fig. 4**).

**Figure 6:**
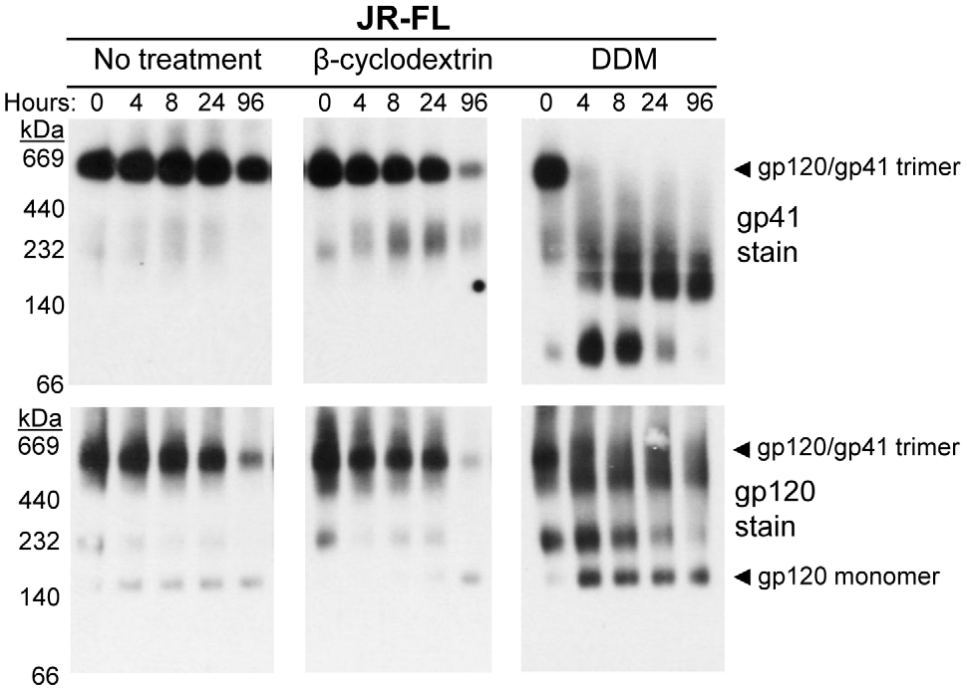
Time course dissociation of HIV-1 Env trimers in the presence of membrane altering reagents visualized using BN-PAGE. HIV-1_LAI-JR-FL_ MC, produced in 293T cells, was incubated for 0 h, 4 h, 8 h, 24 h, or 96 h at 37°C in the presence of no compound (left), 70 mM β-cyclodextrin (cholesterol scavenger; center), or 1% DDM (mild detergent; right). Following incubation for the indicated time periods, samples were analyzed by BN-PAGE and Western blot as in **Fig. 4**.

In order to determine whether the viral membrane - or components thereof - help maintain trimer stability, HIV-1 virions were incubated in the presence or absence of the cholesterol scavenger (2-hydroxypropyl-)β-cyclodextrin [73] or with the mild BN-PAGE detergent, DDM, in a 37°C time-course and Env was monitored using BN-PAGE over 96 h (**Fig. 6**). In the absence of any treatment, virion-associated Env trimers from HIV-1_JR-FL_ only partially dissociated during the time course, as we observed previously with HIV-1_JR2_. In repeated experiments, the presence of β-cyclodextrin at HIV-inactivating concentrations slightly increased the dissociation rate of Env trimers. In the presence of 1% DDM, Env trimers almost completely dissociated by 4 hrs, which is faster than infectivity decays with the corresponding HIV-1_JR-FL_ (t_1/2_∼16 h; data not shown). These data suggest that the presence of the viral membrane helps stabilize native Env trimers at 37°C as viral infectivity decays in an Env-dependent manner, and that cholesterol contributes, albeit modestly, to this effect.

One possible explanation for Env-dependent viral inactivation in absence of complete Env trimer dissociation at 37°C is that the trimer spontaneously assumes a fusion-active state. HIV-1 entry inhibitors C34 and BMS-378806 can block conformational changes in Env required for fusion [22]. Virions that were pulse-treated for 20 h with varying concentrations of each inhibitor at 37°C showed similar rates of viral infectivity decay as untreated virions (data not shown), suggesting that Env trimers inactivate in a manner distinct from that which leads to fusion.

**Figure 7:**
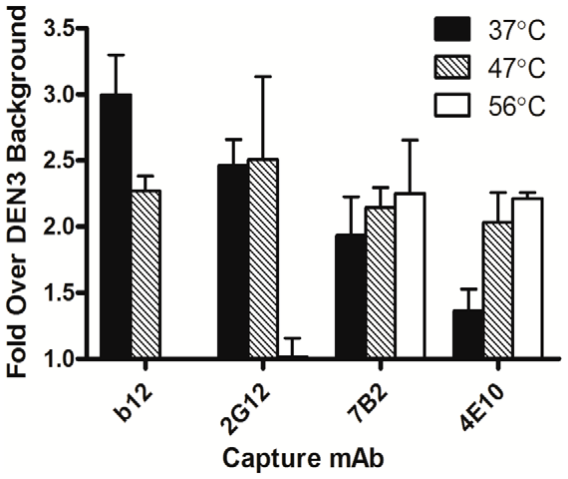
Effect of heat pre-treatment on mAb-virion capture efficiency. HIV-1_LAI-JR-FL_ (MC) virions, produced in 293T cells, were incubated at the indicated temperatures for 1 h and anti-gp120 mAbs 2G12 (outer face), b12 (CD4 binding site), anti-gp41 mAbs 4E10 (MPER), and 7B2 (immunodominant loop), and the irrelevant mAb DEN3 were used to capture the heat treated virions in an in-solution virus capture assay. Amount of virion (p24) equivalents captured were determined using an in-house p24 ELISA and reported relative to background levels captured using DEN3.

To verify that heat-induced dissociation of virion-associated Env trimers observed using BN-PAGE was not a phenomenon that occurred following sample preparation, we used a previously described in-solution virion capture assay to determine efficiency of capture of free virions by anti-Env antibodies prior to, and immediately following heat treatment [10]. We also used HIV-1_JR-FL_ produced using the pLAI backbone vector, which displays well cleaved, relatively homogeneous Env trimers that are recognized less efficiently using non-neutralizing antibody when compared to backbone-matched HIV-1_JR-CSF_ and HIV-1_ADA_ [10]. Indeed, we found that following heat treatment MC HIV-1_JR-FL_ was now captured less efficiently using gp120 antibodies (e.g. b12 and 2G12) but more efficiently by gp41 antibodies (e.g. 7B2 and 4E10), consistent with the notion that native Env trimers had dissociated from free virions, leaving behind fewer gp120 molecules associated with the virus while revealing more gp41 epitopes in the process (**Fig. 7**).

### Env thermostability among acute primary isolates within clades A, B and C

**Figure 8:**
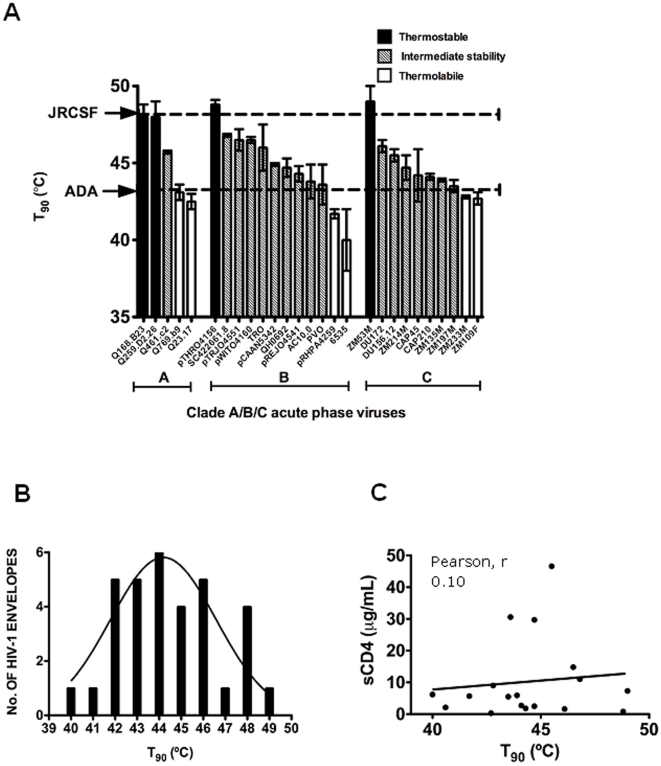
Thermostabilities (T_90_ values) of acute phase standard panel viruses (PSVs) of clades A, B and C, and lack of a relationship with susceptibility to soluble CD4. (**A**) Bar graph indicating the various T_90_ values with that of the thermostable HIV-1_JR-CSF_, and thermolabile HIV-1_ADA_ indicated with dashed lines. Env panel members that are thermostable (T_90_≥48°C), intermediate in thermostability (43°C<T_90_<48°C), and thermolabile (T_90_≤43°C) are indicated by filled, hatched and open bars, respectively. (**B**) Bell curve (normal distribution) in T_90_ values of HIV-1 Envs used in this study, including Envs from acute phase panels of clades A, B and C (Tables 1 and 2), n = 34. Mean, 44.2°C; Std. Dev., 2.4°C; Range, 40.0–49.0°C. The curve-fit (nonlinear regression lorentzian) was made using Prism software (Graphpad, CA). (**C**) Lack of correlation between T_90_ and reported IC_50_ values of soluble CD4 against HIV-1 standard Env panels (clades B and C). Soluble CD4 IC_50_ data are taken from Wu et al [79], excluding resistant isolates (IC_50_>50 µg/ml) for which exact values are undetermined.

HIV-1 Envs ADA, JR-FL and JR-CSF were cloned roughly two decades ago from a monocytotropic virus [74], [75], and from the frontal lobe and cerebrospinal fluid of a patient with severe AIDS encephalopathy [76], respectively. To obtain thermostability data using a broader, more recent and representative sampling of HIV-1, Envs were sourced from three HIV-1 acute phase panels of clades A [59], B and C [57], and T_90_ values were determined (**Table 2**). Some of these acute Envs, such as Q259.d2.26 and Q168.b23 (clade A), pTHRO4156 (clade B), as well as ZM53M (clade C) were relatively thermostable (T_90_≥48°C), similar to HIV-1_JR-CSF_ (**Fig. 8A**). However, heat labile Envs (T_90_≤43°C) were also found, such as Q23.17 (clade A), 6535 and pRHPA4259 (clade B), as well as ZM109F and ZM233M (clade C) (**Fig. 8A**). The distribution of T_90_ values with these Env panels (**Table 2**), and those from **Table 1**, ranges from ∼40 to 50°C, with an overall mean of 44.2°C, and a standard deviation of 2.4°C (**Fig. 8B**). We conclude that thermostability varies significantly among functional Envs of primary HIV-1, as sourced from both acute and chronic natural infection, as well as of different cellular tropisms, clades, tissues and patients.

**Table 2:** Thermostability (T_90_ values) and sensitivity to soluble (4-domain) CD4 of HIV-1 PSVs bearing Envs from acute phase virion panels (clades A, B and C).

Virus	Infectivity^1^	T_90_ ± SEM^2^	sCD4 IC_50_ (µg/ml)^3^
**Clade A**			
Q259.D2.26	++	48.0±1.0	*nd* 4
Q168.B23	++	48.0±0.8	*nd*
Q461.c2	+++	45.7±0.1	*nd*
Q769.b9	+	43.1±0.5	*nd*
Q23.17	+	42.5±0.5	*nd*
**Clade B**			
pTHRO4156	+/−	48.8±1.1	0.8
SC422661.8	++	46.8±0.2	11
pTRJO4551	++	46.5±0.7	>50
pWITO4160	+/−	46.5±0.2	14.8
TRO	+/−	46.0±1.5	>50
pCAAN5342	+++	44.9±0.1	>50
QH0692	+/−	44.7±0.6	2.5
pREJO4541	++	44.3±0.5	1.8
AC10.0	++	43.8±1.1	>50
PVO	++	43.6±1.3	30.6
pRHPA4259	+/−	41.7±0.3	5.7
6535	+	40.0±2.0	6.2
**Clade C**			
ZM53M	++	49.0±1.0	7.3
DU172	+/−	46.1±0.4	1.6
DU156.12	++	45.5±0.4	46.6
CAP210	++	45.5±0.3	2.7
ZM214M	++	44.7±0.8	29.7
CAP45	++	44.2±1.5	2.1
ZM135M	++	43.9±0.1	5.9
ZM197M	+/−	43.5±0.4	5.5
ZM233M	+/−	42.8±0.1	9.0
ZM109F	+++	42.7±0.4	0.3

1 Infectivities were determined in TZM-bl cells and the resulting relative light units (RLUs) were categorized as follows: +/−, 80,000–149,999; +, 150,000–349,999; ++, 350,000–999,999; +++, ≥1,000,000.

2 T_90_, the temperature in °C at which HIV-1 infectivity decreases by 90% in 1 h, and the standard error of the mean (SEM) of at least three independent experiments.

3 sCD4 IC_50_ data taken from ref. [79].

4 *nd*, not determined.

### Effect of point mutations in the MPER of Env on thermostability

**Figure 9:**
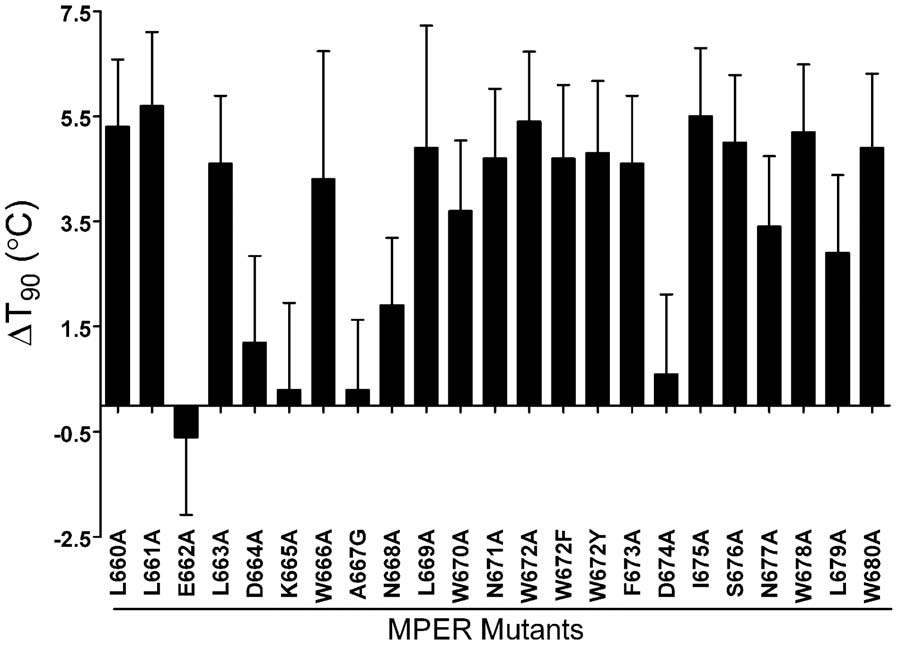
Change in thermostability of HIV-1_JR2_ (ΔT_90_ values) due to Ala mutations in the MPER of gp41. T_90_ values were determined for each Ala mutant and for parental HIV-1_JR2_ and the ΔT_90_ values were calculated (ΔT_90_ = T_90_ of parental−T_90_ of mutant).

The MPER forms a stalk at the base of the trimer, and abuts the membrane, which we in turn have shown above to contribute to trimer stability. We have described previously a panel of MPER Ala mutants of HIV-1_JR2_ [56], and decided to test the thermostability (T_90_s) of these mutants. Interestingly, the majority of the MPER Ala mutants were destabilizing (**Fig. 9**). Without exception, mutation to Ala of the hydrophobic residues in this region (Trp, Phe, Leu, Ile) was extremely destabilizing (4–5°C decrease in T_90_), whereas mutation of charged residues (Glu, Lys, Asp) had little effect. For one of the least stable mutants, W672A, we further measured its stability at 37°C and found that infectivity of the mutant decayed more rapidly than wildtype as well (t_1/2_ = 6.3 h *versus* 16 h). Substitutions in the MPER have been shown previously to affect Env incorporation into virions [77]. This made BN-PAGE analysis more difficult with mutant W672A; however, Env trimers of mutant W672A also appear to dissociate more rapidly than wildtype (data not shown). Overall, the MPER of gp41 appears to be crucial for maintaining stability of native Env trimers of HIV-1.

### HIV-1 sensitivities to CD4 and heat are distinct

CD4 specifically primes Env trimers for fusion and can promote shedding of gp120, particularly with TCLA strains of HIV-1 [18], [19], [20], [21]. Although Env from the TCLA strain HIV-1_LAI_ is sensitive to shedding of gp120 induced by soluble CD4 and to neutralizing antibody, we noticed that this strain was more thermostable than some primary isolates (**Tables 1** **and** **2**). Env spike stability has previously been shown not to predict neutralization sensitivity [78]. Thus, for the HIV-1 panel members we plotted cognate T_90_ values against previously reported IC_50_s of sCD4 [79]. As expected, no linear correlation or other obvious relationship was found between Env T_90_ values and IC_50_s of sCD4 (R = 0.10; **Fig. 8C**). We also performed the heat gradient assay using HIV-1 Envs JR-CSF, JR-FL, and ADA in the presence of sCD4 at concentrations close to the IC_50_. In this format, we observed at most only modest changes in T_90_ values due to the presence of sCD4 (ΔT_90_≤1°C), indicating that sCD4 may slightly sensitize Env to heat but that the effects of heat and sCD4 on HIV-1 infectivity are not strongly cooperative (**Table 3**).

**Table 3:** Effect of the presence of soluble CD4 on the thermostability (T_90_) of HIV-1.

HIV-1 Env^1^	sCD4 IC_50_ 2 (µg/ml)	ΔT90 (°C)^3^ in the presence of the indicated concentrations of sCD4:	
		0.3 µg/ml	1.2 µg/ml
JR-CSF	1.1	−0.5	−0.5
JR-FL	1.2	−0.1	−0.8
ADA	0.17	−1.0	*nd*

1 HIV-1 (LAI chimeric MCs) produced in 293T cells.

2 Concentration of 4-domain soluble CD4 (sCD4) required to decrease HIV-1 infectivity by 50%.

3 ΔT_90_ = (T_90_ in the presence of sCD4)−(T_90_ in the absence of sCD4). HIV-1 was treated with the indicated concentrations of sCD4 and immediately subjected to a range of temperatures for 1 h. Infectivity was measured in TZM-bl cells. *nd*, not determined due to insufficient infectivity at the indicated concentration of sCD4.

### Functional decay of select Envs at physiological temperature (37°C)

**Figure 10:**
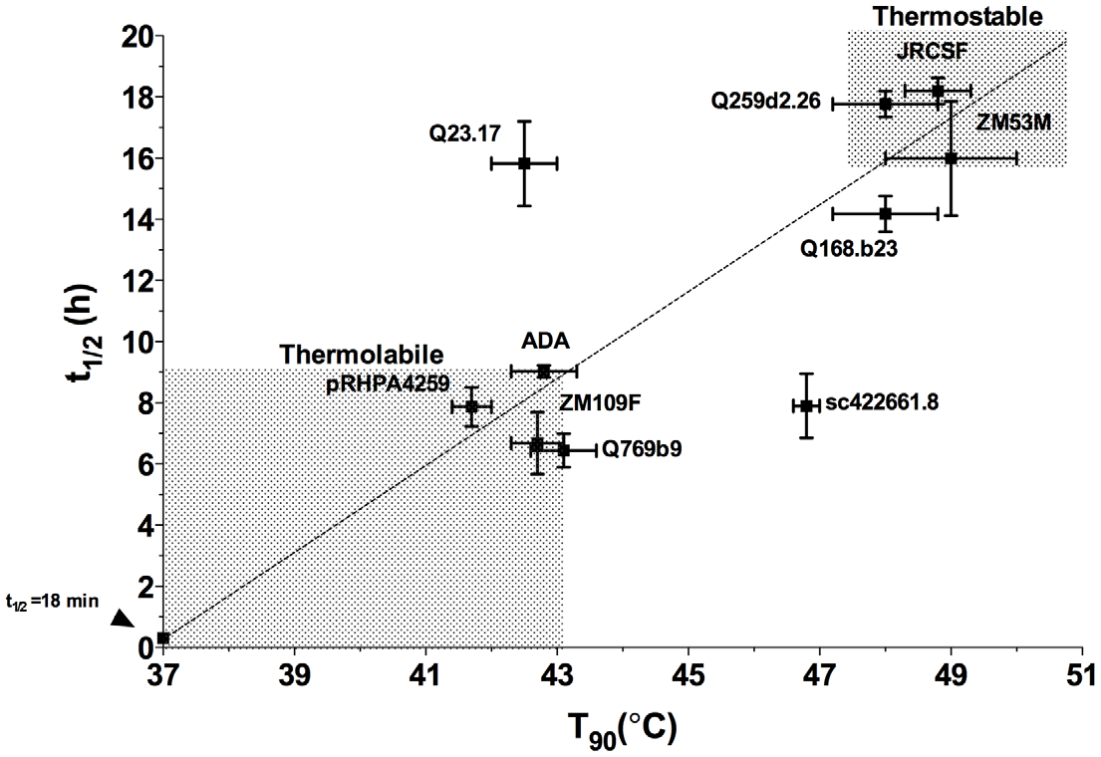
Relationship between thermostability (T_90_) and infectivity half-life at 37°C (t_1/2_) for HIV-1 (PSVs) from clades A, B and C. Pearson, r = 0.686; P value (2-tailed) = 0.0286 (significant). Identity of Env is shown beside each point. For emphasis, shaded regions designate areas of thermolabile and thermostable Envs. The arrow to the left of the origin indicates the point at which t_1/2_ = 18 min, which, at 37°C, also corresponds to a T_90_ = 37°C.

To further test the possible physiological relevance of our thermostability measurements, HIV-1 variant Envs with extreme thermostabilities across the three clades (*i.e.* 43.1≥T_90_s≥46.8°C; n = 10) were examined for infectivity decay at 37°C. Here, we observed a range in half-lives (t_1/2_) from 6.4–18.2 h (**Table 4**), and found that a positive correlation existed between T_90_ and t_1/2_ values for the 10 pseudotyped viruses tested (**Fig. 10**; r (Pearson) = 0.686, P value = 0.029), with Envs of isolates Q23.17 and sc422661.8 being the two main outliers. Significantly, we were able to identify Envs that are generally thermostable (*i.e.* T_90_≥48°C and t_1/2_≥16 h with Envs Q259d2.26, JR-CSF and ZM53M) as well as those that are generally thermolabile (*i.e.* T_90_≤43°C and t_1/2_≤9 h with Envs Q769b9, ADA/pRHPA4259 and ZM109F) from clades A, B and C, respectively.

**Table 4:** Thermostability (T_90_) and half-life of infectivity decay at 37°C (t_1/2_) of select HIV-1 (PSVs) from clades A, B and C.

Virus (clade)	Infectivity^1^	T_90_ ± SEM (°C)^2^	t_1/2_ ± SEM (h)^3^
Q259d2.26 (A)	++	48.0±0.8	17.8±1.1
Q168b23 (A)	++	48.0±0.8	14.2±1.8
Q769b9 (A)	+	43.1±0.5	6.4±1.3
Q23.17 (A)	+	42.5±0.5	15.8±2.4
JRCSF (B)	++	48.8±0.5	18.2±0.7
ADA (B)	++	42.8±0.5	9.0±0.4
sc422661.8 (B)	++	46.8±0.2	7.9±2.1
pRHPA4259 (B)	+/−	41.7±0.3	7.9±1.3
ZM53M (C)	++	49.0±1.0	16.0±3.7
ZM109F (C)	+	42.7±0.4	6.7±2.0
		Mean: 45.3	Mean: 12.0

1 Infectivities were determined in TZM-bl cells and the resulting relative light units (RLUs) were categorized as follows: +/−, 100,000–149,999; +, 150,000–349,999; ++, 350,000–999,999; +++, ≥1,000,000.

2 T_90_, the temperature at which HIV-1 infectivity decreases by 90% in 1 h, and the standard error of the mean (SEM) of at least three independent experiments.

3 t_1/2_, the time (in hours) at which HIV-1 infectivity decreases by 50% under physiological conditions (37°C), and the standard error of the mean (SEM) of at least three independent experiments.

### Intersubunit disulfide bond (“SOS”) enhances Env trimer thermostability

**Figure 11:**
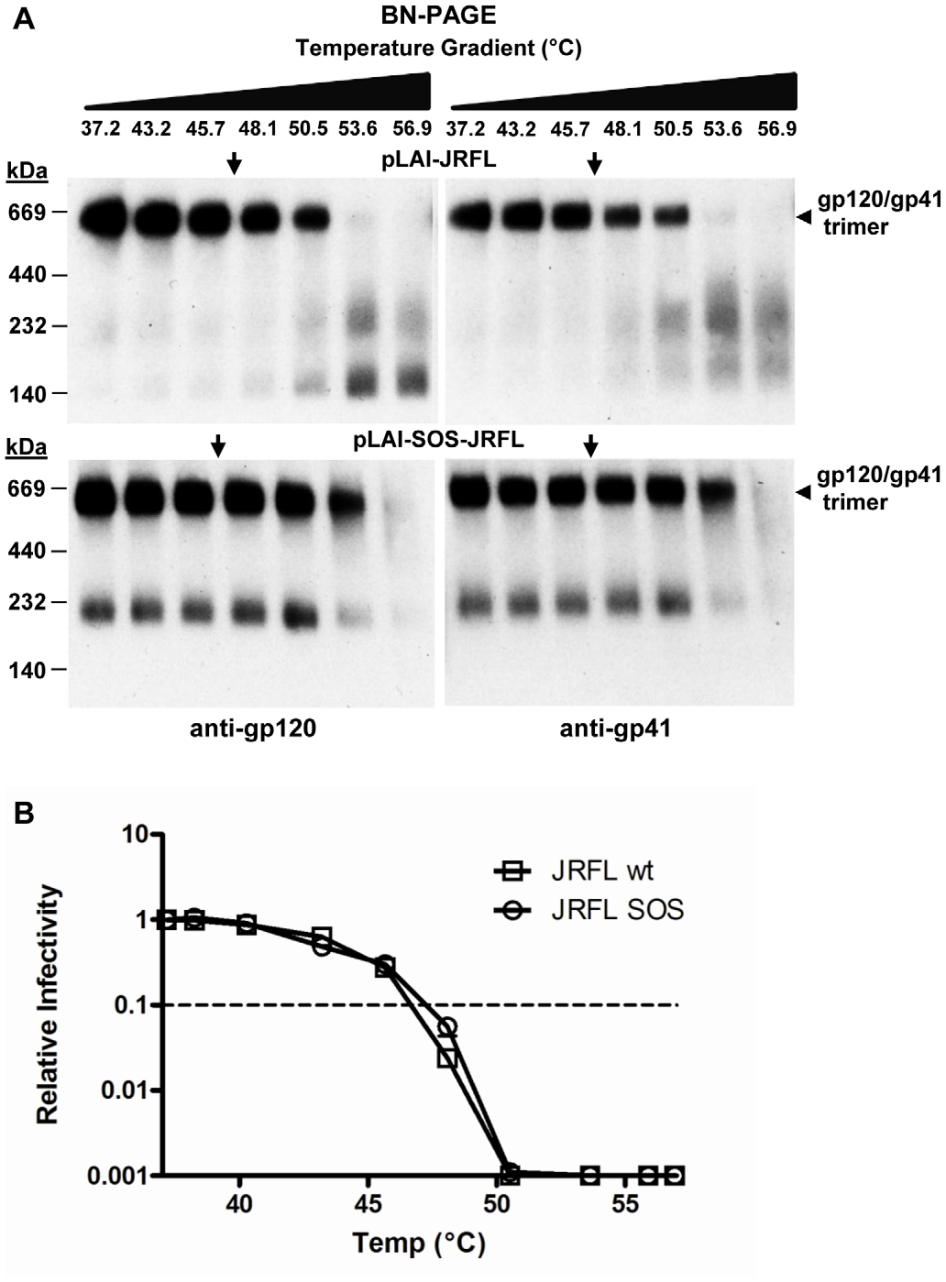
Intersubunit disulfide bond (“SOS”) increases apparent thermostability of trimeric Env but does not increase thermostability of infectivity (T_90_) of HIV-1_LAI-JR-FL_. (**A**) BN-PAGE analysis of the thermostability of wildtype HIV-1_LAI-JR-FL_ and SOS-HIV-1_LAI-JR-FL_, incubated as whole virions for 1 hr at indicated temperatures, then prepared for BN-PAGE and subsequent blotting using mAb cocktails to gp120 and gp41. (**B**) Thermostability comparison in the infectivity assay of wildtype HIV-1_LAI-JR-FL_ and SOS-HIV-1_LAI-JR-FL_, using the same heat gradient as in panel A.

Introduction of two cysteine substitutions (*i.e.* A501C and T605C) has previously been shown to stabilize Env in a soluble, truncated form of the trimer [46]. As the cognate virus, HIV-1_JR-FL-SOS_, infects host cells upon addition of a reducing reagent (e.g. dithiothreitol, DTT) [80], we wished to determine whether the SOS disulfide might also stabilize virion-associated, full-length Env trimers. We introduced the SOS mutations into full-length Env in the HIV-1_JR-FL-LAI_ MC backbone, and, using BN-PAGE analysis, examined wt and SOS Env from virion preparations that were normalized for Env content. The SOS disulfide was found to enhance the stability of virion-associated trimeric Env, as the corresponding band faded at temperatures ∼3°C higher than that of the wildtype trimeric Env (**Fig. 11A**). However, despite the trimer stabilizing effect of the SOS disulfide, no increase was observed in the T_90_ of SOS relative to wildtype virions (**Fig. 11B**).

## Discussion

In the present study, we determined specific stabilities of functional Env trimers of HIV-1, which should help in establishing structure-function relationships and other biological implications of Env stability. Among the methods used to assay Env stability, we found the thermogradient infectivity (T_90_) assay to be the most rapid, high in throughput, facile, and precise. Ten Envs with extreme T_90_ values were also examined for Env decay at physiological temperature (t_1/2_), which yielded a positive correlation. However, Envs from isolates Q23.17 and sc422661.8 deviated from this correlation. Such discrepancies may result from a tendency of certain Envs to unfold particularly rapidly within specific temperature ranges [81]. Additionally, temperature-dependent factors in the extrinsic medium (e.g. cofactors or enzymes) may inactivate certain Envs more rapidly than others at physiological temperature, and still other explanations might exist. Hence, multiple different measures of Env stability under physiological conditions and other methods to probe the Env oligomer (e.g. BN-PAGE and virus capture assays) are recommended if the goal is to identify native Envs of broad stability or with specific stability profiles.

We highlight several elements necessary to make determinations of thermostability (T_90_) and other stability measurements of functional Env reproducible. First, use of cloned Envs removes uncertainty associated with undefined quasispecies. Second, HIV-1 backbone constancy minimizes assay variability contributed by non-Env virion components. Though we found that backbones and RTs of both clades A and B had remarkably similar thermostabilities, certain RTs of HIV-1 Group O reportedly have unusually high thermostability [82]. Third, a gradient PCR block provides accurate temperatures and the ability to multiplex. Fourth, using only measurements in the linear part of the infectivity curve with non-saturating amounts of virus is crucial to mitigating error. Fifth, HIV-1 produced in a defined cell line overcomes possible effects of different producer cells on Env stability and heterogeneity. In our hands, 293T producer cells yielded more uniform results than MT-2/CCR5ΔCT cells. Virion heterogeneity may indeed arise from asynchronous virus replication, heterogeneity in the producer cells, and/or exposure of nascent virions to cell receptors that may promote destabilization of Env. We note that LAI-chimeric MCs JR-CSF and SF162 were produced in PBMCs and found to have T_90_ values comparable to that produced in MT-2/CCR5ΔCT cells (NA, MBZ, unpublished observations). Nevertheless, producer cell, virus stock age, and other factors may affect intrinsic stability or copy number of fusion-competent Env spikes on the virus [83], [84]. We suggest that Env stability assays be individually validated. Our results using widely available Envs and Env standard panels [57] should help in this regard.

BN-PAGE analyses lent further evidence of stability differences in Env trimers for ADA, JR-FL and JR-CSF. Env trimer bands tended to disappear on BN-PAGE Western blot at pre-incubation temperatures a few degrees above the cognate T_90_. The lag in temperature might be due, at least in part, to the presence of other heat labile HIV-1 components required for infectivity (see below). However, our present study suggests that Env is often limiting in HIV-1 inactivation. We have previously shown that oligomers of uncleaved gp160 can be resistant to heat induced dissociation [10], so unprocessed Env, non-functional but relatively stable Env trimers, or other heterogeneity in processed Env may confound analyses of functional Env trimers on BN-PAGE. A further possibility is that heat may alter the conformation of Env trimers subtly, rendering them inactive but difficult to distinguish using standard BN-PAGE. This explanation may also relate to the persistence of Env trimers on BN-PAGE after incubation at 37°C for four days, even though infectivity was nearly abrogated under these conditions. Of note with BN-PAGE and capture assays, we generally avoided PSVs and focused on intact MCs as we have found that these are less contaminated with uncleaved Env [10]. Using both assays we have shown that heat exposes gp41 (BN-PAGE) and promotes virion capture by (non-neutralizing) mAb 7B2 against the immunodominant region of gp41, both results of which are consistent with gp120 shedding.

We did not attempt to quantify all possible contributions from different virion components to HIV-1 thermostability. We did however measure RT thermostability and found it to be more stable than Env trimers, consistent with experiments by others [82], [85]. Above ∼50°C membranes may become unstable [86], which might also help to explain greater dissociation of Env trimers and loss of infectivity at elevated temperatures *versus* physiological temperature. Indeed, we found that treatment of virions with β-cyclodextrin - which blocks viral infection primarily by removing essential cholesterol from the membrane [73] - slightly increased the rate of trimer dissociation. Moreover, complete solubilization of Env in mild detergent led to rapid dissociation. Other thermolabile components may include the virion matrix, capsid and the RNA dimer [87], [88]. If the half-life of native Env (t_E_), and that of the HIV backbone (t_HB_), are designated as the two main contributors to HIV-1 infectivity decay, the infectivity half-life of the whole particle (t_1/2(HIV-1)_) can be described using the ‘harmonic mean’ of the component parts: t_1/2(HIV-1)_ = (t_E_*t_HB_)/(t_E_+t_HB_). Thus, for labile Envs (*i.e.* t_E_«t_HB_), t_E_ has the most weight in determining t_1/2(HIV-1)_. Conversely, when Env is stable (*i.e.* t_E_»t_HB_), t_1/2(HIV-1)_ approaches the half-life of the backbone, t_HB_. Thus, heat gradient/infectivity assays may be less sensitive to measurement of T_90_ and t_1/2_ (37°C) values beyond ∼48–50°C and ∼20 h, respectively, due to inactivation of the viral backbone. Screening more virus backbones may help resolve this issue.

HIV-1_ADA_ was shown to be more susceptible than HIV-1_JR-CSF_ to GuHCl. We found that GuHCl treated and washed (pelleted) virions showed faded Env trimer bands by BN-PAGE (unpublished observations, ER, DPL, MBZ), suggesting that Env trimers were forced to dissociate. However, unlike heat and GuHCl, urea and pH changes affected ADA and JR-CSF equally. The nature of the discrepancy in activity of the two denaturants is unknown, but might relate to a hyper-sensitivity of ADA to the presence of charged ions with the GuHCl salt that are absent with urea, a non-ionic chaotrope. A recent study identified a substitution in the inner domain of gp120 (H66N) that alleviated a “cold labile” phenotype attributed to Env of HIV-1_ADA_ [81]. In that study, ADA was found to inactivate over several days at 4°C, due in part to exposure of cryptic sites on gp41. Indeed, using BN-PAGE we have observed gp41 dissociation product with GuHCl-treated HIV-1 (DPL, MBZ; unpublished observations). Future experiments will focus on functional Env stability under various destabilizing conditions in combination with Env mutagenesis to identify and understand differences in sensitivity of Env to spontaneous and induced inactivation (DPL, MBZ; manuscript in preparation). Overall, our data show that Env can be irreversibly inactivated in ways that are specific, a feature reminiscent of Env sensitivity to irreversible inactivation by different entry inhibitors [89].

The majority of HIV-1 Envs tested from clades A, B and had thermostabilities within ∼2°C of the mean T_90_, 44.2°C, suggesting a selective pressure *in vivo* that tightly regulates Env trimer stability. As different parameters of protein stability often can correlate [90], [91], it is possible that thermolabile HIV-1 Env variants will also be less fit *in vivo* due to more rapid infectivity decay in portals of viral entry. Unstable Envs might be more susceptible to elevated temperatures during fever, mechanical stresses encountered during viral trafficking such as transcytosis or trans-display on cells like DCs, and to destabilizing ligands including antibody [92], [93]. Envs that are generally more stable will tend to fare better in such situations; however, undergoing fusion may become increasingly difficult. Gp120 shedding from labile spikes has been suggested to contribute to pathogenesis and immune dysregulation, although the significance of these effects is controversial. Finally, stability of Env may affect adaptive immune responses in unknown and likely complex ways involving Env quaternary structure, Env processing, post-translational modifications such as glycosylation, T-cell epitope peptide processing rates, and other parameters affecting Env immunogenicity. In our study, we observed no significant difference in Env thermostability between viruses from acute and chronic infection; however, it might be interesting to explore more deeply the specific stabilities of Env during acquisition of infection and disease progression. Structural elements contributing to the narrow range in Env thermostability might be broadly conserved; however, multiple variable elements may also co-adapt to preserve Env fitness. Point mutagenesis cannot easily address non-additive contributions from many distal regions of Env. For example, JR-CSF and ADA Envs have 9.1% sequence divergence, including in V1V2, V3, V4, V5, C1, and C5, which makes it difficult if not impossible to pinpoint single residues responsible for the different stability phenotypes. Nevertheless, mutagenesis and Env structure-function studies, in combination with bioinformatic tools, may help deconstruct Env trimer stability, as would determination of Env trimer structure in high resolution.

Mutations in many different regions of Env affect retention of gp120 on virions or cell surfaces displaying HIV-1 Env, such as those in the inner domain of gp120 and the central ectodomain of gp41 [50], [94], [95], [96]. We confirmed some of these findings with a few mutants in our thermostability experiments (our unpublished observations; ER, MBZ). Of recent interest, Ruprecht et al have shown that neutralizing mAbs to the MPER of gp41 can destabilize Env trimers [93]. Our Ala scan analysis now shows that the MPER is also a determinant of trimer stability on unliganded spikes. While the MPER is not thought to directly interact with gp120 in the native trimer, it likely influences interactions between gp41 subunits and the viral membrane [41], [94], [97], [98]. We found that mutation of the hydrophobic residues in the MPER had the most destabilizing effects. In NMR/EPR studies of membrane-embedded MPER peptides these hydrophobic residues are most deeply immersed in the membrane [41], and in a crystal structure of a homotrimeric MPER peptide these same residues are buried in the trimer interface [40]. The importance of the MPER in trimer stability may also be linked to our observation that the viral membrane plays an important role in stabilizing native Env, and that the MPER can interact specifically with cholesterol and other membrane lipids [98], [99]. That the MPER is crucial for Env stability, Env incorporation into virions and membrane perturbation during fusion also helps to explain its remarkable sequence conservation [98]. For the design of Env-based vaccines, the MPER and other trimer determining regions can be targeted to help stabilize Env mimetics in native form without the use of stabilizing techniques that interrupt Env function (*e.g.* cleavage site knockouts or added disulfides) that alter the antigenic structure of Env.

We found no clear relationship between Env trimer thermostability and sensitivity of HIV-1 to soluble CD4 or neutralizing antibodies with clade B panel members (IC_50_s) (data not shown; [57]). However, lack of such relationships was not unexpected. Prior studies have found little correlation between neutralization sensitivity with particular antibodies and sensitivity to soluble CD4, Env copy number or retention of gp120 on HIV-1 PSVs [78], [79]. Studies have also shown that sCD4 will labilize certain primary Envs more than others [22], [100], which is explained at least in part by the ability of structural layers in gp41 to facilitate conformational transitions upon CD4-binding and that control the stability between gp120 and gp41 [45], [101], [102]. In our study, destabilizing effects of soluble CD4 and heat showed little synergy; however, it may be possible to relate thermostability of CD4-activated Env using more specific assay formats. Moreover, it is possible that certain mutations in Env may simultaneously affect trimer stability as well as sensitivity to neutralizing antibody through changes to fusion efficiency or cell receptor recognition [103], [104]. Finally, certain antibodies may themselves be destabilizing to Env trimers, as has been demonstrated previously using the b12 neutralizing antibody [105] and more recently using MPER antibodies [93].

Eliciting a particular neutralizing antibody response that is specific for native Env will likely be difficult if it is prone to dissociate and the byproducts are structurally distinct. Unlike natural infection in which progeny virions displaying freshly made trimers are constantly replenishing decayed ones, non-native byproducts of Env spike decay will accumulate with vaccines administered by bolus injection. These byproducts may divert B cells with cross-reactive B cell receptors (BCRs) away from native trimers, particularly when present on the same particle [8], [9]. Moreover, gp120, gp41 six-helix bundles, as well as unprocessed gp160 may be more stable than native Env and can persist in lymph nodes for many months [106], [107]. This scenario may be exacerbated by the low copy number of native trimers that are sparsely distributed on virions [108], [109]. We found evidence of physical dissociation of Env trimers after 4 days (96 h) at physiological temperature. At this rate *in vivo*, trimer dissociation or other forms of Env decay may or may not be enough to impact B cell affinity maturation that develops in germinal centers over several days [110]. However, we have shown that Env dependent inactivation at 37°C (t_1/2_) of HIV-1 can be rapid (e.g. as low as 6 h and presumably this can go lower still). The rate of decay with HIV-1 Env that is presented to pertinent immune cells *in vivo* is poorly understood [111], [112], but it is certainly possible that the stability of native Env may influence elicitation of neutralizing antibody. Further study of the biological consequences of HIV-1 Env spike stability is therefore warranted, as is determining whether it can be manipulated to elicit favorable neutralizing antibody responses through immunization.

## References

[1] Stamatatos L, Morris L, Burton DR, Mascola JR (2009). Neutralizing antibodies generated during natural HIV-1 infection: good news for an HIV-1 vaccine?. Nat Med.

[2] Barouch DH (2008). Challenges in the development of an HIV-1 vaccine.. Nature.

[3] Karlsson Hedestam GB, Fouchier RA, Phogat S, Burton DR, Sodroski J (2008). The challenges of eliciting neutralizing antibodies to HIV-1 and to influenza virus.. Nat Rev Microbiol.

[4] Zwick MB, Burton DR (2007). HIV-1 neutralization: mechanisms and relevance to vaccine design.. Curr HIV Res.

[5] Fouts TR, Binley JM, Trkola A, Robinson JE, Moore JP (1997). Neutralization of the human immunodeficiency virus type 1 primary isolate JR-FL by human monoclonal antibodies correlates with antibody binding to the oligomeric form of the envelope glycoprotein complex.. J Virol.

[6] Yang X, Wyatt R, Sodroski J (2001). Improved elicitation of neutralizing antibodies against primary human immunodeficiency viruses by soluble stabilized envelope glycoprotein trimers.. J Virol.

[7] Sattentau QJ, Moore JP (1995). Human immunodeficiency virus type 1 neutralization is determined by epitope exposure on the gp120 oligomer.. J Exp Med.

[8] Poignard P, Moulard M, Golez E, Vivona V, Franti M (2003). Heterogeneity of envelope molecules expressed on primary human immunodeficiency virus type 1 particles as probed by the binding of neutralizing and nonneutralizing antibodies.. J Virol.

[9] Moore PL, Crooks ET, Porter L, Zhu P, Cayanan CS (2006). Nature of nonfunctional envelope proteins on the surface of human immunodeficiency virus type 1.. J Virol.

[10] Leaman DP, Kinkead H, Zwick MB (2010). In-solution virus capture assay helps deconstruct heterogeneous antibody recognition of human immunodeficiency virus type-1.. J Virol.

[11] McCune JM, Rabin LB, Feinberg MB, Lieberman M, Kosek JC (1988). Endoproteolytic cleavage of gp160 is required for the activation of human immunodeficiency virus.. Cell.

[12] Leonard CK, Spellman MW, Riddle L, Harris RJ, Thomas JN (1990). Assignment of intrachain disulfide bonds and characterization of potential glycosylation sites of the type 1 recombinant human immunodeficiency virus envelope glycoprotein (gp120) expressed in Chinese hamster ovary cells.. J Biol Chem.

[13] Murakami T (2008). Roles of the interactions between Env and Gag proteins in the HIV-1 replication cycle.. Microbiol Immunol.

[14] Klatzmann D, Champagne E, Chamaret S, Gruest J, Guetard D (1984). T-lymphocyte T4 molecule behaves as the receptor for human retrovirus LAV.. Nature.

[15] Dalgleish AG, Beverley PC, Clapham PR, Crawford DH, Greaves MF (1984). The CD4 (T4) antigen is an essential component of the receptor for the AIDS retrovirus.. Nature.

[16] Lusso P (2006). HIV and the chemokine system: 10 years later.. EMBO J.

[17] Gallo SA, Finnegan CM, Viard M, Raviv Y, Dimitrov A (2003). The HIV Env-mediated fusion reaction.. Biochim Biophys Acta.

[18] Moore JP, McKeating JA, Weiss RA, Sattentau QJ (1990). Dissociation of gp120 from HIV-1 virions induced by soluble CD4.. Science.

[19] Orloff SL, Kennedy MS, Belperron AA, Maddon PJ, McDougal JS (1993). Two mechanisms of soluble CD4 (sCD4)-mediated inhibition of human immunodeficiency virus type 1 (HIV-1) infectivity and their relation to primary HIV-1 isolates with reduced sensitivity to sCD4.. J Virol.

[20] Fu YK, Hart TK, Jonak ZL, Bugelski PJ (1993). Physicochemical dissociation of CD4-mediated syncytium formation and shedding of human immunodeficiency virus type 1 gp120.. J Virol.

[21] Moore JP, McKeating JA, Huang YX, Ashkenazi A, Ho DD (1992). Virions of primary human immunodeficiency virus type 1 isolates resistant to soluble CD4 (sCD4) neutralization differ in sCD4 binding and glycoprotein gp120 retention from sCD4-sensitive isolates.. J Virol.

[22] Haim H, Si Z, Madani N, Wang L, Courter JR (2009). Soluble CD4 and CD4-mimetic compounds inhibit HIV-1 infection by induction of a short-lived activated state.. PLoS Pathog.

[23] Chertova E, Bess JW, Crise BJ, Sowder IR, Schaden TM (2002). Envelope glycoprotein incorporation, not shedding of surface envelope glycoprotein (gp120/SU), is the primary determinant of SU content of purified human immunodeficiency virus type 1 and simian immunodeficiency virus.. J Virol.

[24] Herrera C, Spenlehauer C, Fung MS, Burton DR, Beddows S (2003). Nonneutralizing antibodies to the CD4-binding site on the gp120 subunit of human immunodeficiency virus type 1 do not interfere with the activity of a neutralizing antibody against the same site.. J Virol.

[25] Burrer R, Haessig-Einius S, Aubertin AM, Moog C (2005). Neutralizing as well as non-neutralizing polyclonal immunoglobulin (Ig)G from infected patients capture HIV-1 via antibodies directed against the principal immunodominant domain of gp41.. Virology.

[26] Yuan W, Craig S, Yang X, Sodroski J (2005). Inter-subunit disulfide bonds in soluble HIV-1 envelope glycoprotein trimers.. Virology.

[27] Pancera M, Wyatt R (2005). Selective recognition of oligomeric HIV-1 primary isolate envelope glycoproteins by potently neutralizing ligands requires efficient precursor cleavage.. Virology.

[28] Dey AK, David KB, Ray N, Ketas TJ, Klasse PJ (2008). N-terminal substitutions in HIV-1 gp41 reduce the expression of non-trimeric envelope glycoproteins on the virus.. Virology.

[29] Herrera C, Klasse PJ, Michael E, Kake S, Barnes K (2005). The impact of envelope glycoprotein cleavage on the antigenicity, infectivity, and neutralization sensitivity of Env-pseudotyped human immunodeficiency virus type 1 particles.. Virology.

[30] Burton DR, Pyati J, Koduri R, Sharp SJ, Thornton GB (1994). Efficient neutralization of primary isolates of HIV-1 by a recombinant human monoclonal antibody.. Science.

[31] Trkola A, Purtscher M, Muster T, Ballaun C, Buchacher A (1996). Human monoclonal antibody 2G12 defines a distinctive neutralization epitope on the gp120 glycoprotein of human immunodeficiency virus type 1.. J Virol.

[32] Walker LM, Phogat SK, Chan-Hui PY, Wagner D, Phung P (2009). Broad and potent neutralizing antibodies from an African donor reveal a new HIV-1 vaccine target.. Science.

[33] Muster T, Steindl F, Purtscher M, Trkola A, Klima A (1993). A conserved neutralizing epitope on gp41 of human immunodeficiency virus type 1.. J Virol.

[34] Zwick MB, Labrijn AF, Wang M, Spenlehauer C, Saphire EO (2001). Broadly neutralizing antibodies targeted to the membrane-proximal external region of human immunodeficiency virus type 1 glycoprotein gp41.. J Virol.

[35] Wu X, Yang ZY, Li Y, Hogerkorp CM, Schief WR (2010). Rational design of envelope surface identifies broadly neutralizing human monoclonal antibodies to HIV-1.. Science.

[36] Zanetti G, Briggs JA, Grunewald K, Sattentau QJ, Fuller SD (2006). Cryo-Electron Tomographic Structure of an Immunodeficiency Virus Envelope Complex In Situ.. PLoS Pathog.

[37] Zhu P, Winkler H, Chertova E, Taylor KA, Roux KH (2008). Cryoelectron tomography of HIV-1 envelope spikes: further evidence for tripod-like legs.. PLoS Pathog.

[38] Liu J, Bartesaghi A, Borgnia MJ, Sapiro G, Subramaniam S (2008). Molecular architecture of native HIV-1 gp120 trimers.. Nature.

[39] Wu SR, Loving R, Lindqvist B, Hebert H, Koeck PJ (2010). Single-particle cryoelectron microscopy analysis reveals the HIV-1 spike as a tripod structure.. Proc Natl Acad Sci U S A.

[40] Liu J, Deng Y, Dey AK, Moore JP, Lu M (2009). Structure of the HIV-1 gp41 membrane-proximal ectodomain region in a putative prefusion conformation.. Biochemistry.

[41] Sun ZY, Oh KJ, Kim M, Yu J, Brusic V (2008). HIV-1 broadly neutralizing antibody extracts its epitope from a kinked gp41 ectodomain region on the viral membrane.. Immunity.

[42] Wyatt R, Kwong PD, Desjardins E, Sweet RW, Robinson J (1998). The antigenic structure of the HIV gp120 envelope glycoprotein.. Nature.

[43] Kwong PD, Wyatt R, Sattentau QJ, Sodroski J, Hendrickson WA (2000). Oligomeric modeling and electrostatic analysis of the gp120 envelope glycoprotein of human immunodeficiency virus.. J Virol.

[44] Helseth E, Olshevsky U, Furman C, Sodroski J (1991). Human immunodeficiency virus type 1 gp120 envelope glycoprotein regions important for association with the gp41 transmembrane glycoprotein.. J Virol.

[45] Leavitt M, Park EJ, Sidorov IA, Dimitrov DS, Quinnan GV (2003). Concordant modulation of neutralization resistance and high infectivity of the primary human immunodeficiency virus type 1 MN strain and definition of a potential gp41 binding site in gp120.. J Virol.

[46] Binley JM, Sanders RW, Clas B, Schuelke N, Master A (2000). A recombinant human immunodeficiency virus type 1 envelope glycoprotein complex stabilized by an intermolecular disulfide bond between the gp120 and gp41 subunits is an antigenic mimic of the trimeric virion-associated structure.. J Virol.

[47] Chen B, Vogan EM, Gong H, Skehel JJ, Wiley DC (2005). Structure of an unliganded simian immunodeficiency virus gp120 core.. Nature.

[48] Wyatt R, Desjardin E, Olshevsky U, Nixon C, Binley J (1997). Analysis of the interaction of the human immunodeficiency virus type 1 gp120 envelope glycoprotein with the gp41 transmembrane glycoprotein.. J Virol.

[49] Poumbourios P, Maerz AL, Drummer HE (2003). Functional evolution of the HIV-1 envelope glycoprotein 120 association site of glycoprotein 41.. J Biol Chem.

[50] Jacobs A, Sen J, Rong L, Caffrey M (2005). Alanine scanning mutants of the HIV gp41 loop.. J Biol Chem.

[51] Wei X, Decker JM, Liu H, Zhang Z, Arani RB (2002). Emergence of resistant human immunodeficiency virus type 1 in patients receiving fusion inhibitor (T-20) monotherapy.. Antimicrob Agents Chemother.

[52] Connor RI, Chen BK, Choe S, Landau NR (1995). Vpr is required for efficient replication of human immunodeficiency virus type-1 in mononuclear phagocytes.. Virology.

[53] Rainwater SM, Wu X, Nduati R, Nedellec R, Mosier D (2007). Cloning and characterization of functional subtype A HIV-1 envelope variants transmitted through breastfeeding.. Curr HIV Res.

[54] Helseth E, Kowalski M, Gabuzda D, Olshevsky U, Haseltine W (1990). Rapid complementation assays measuring replicative potential of human immunodeficiency virus type 1 envelope glycoprotein mutants.. J Virol.

[55] Zwick MB, Wang M, Poignard P, Stiegler G, Katinger H (2001). Neutralization synergy of human immunodeficiency virus type 1 primary isolates by cocktails of broadly neutralizing antibodies.. J Virol.

[56] Zwick MB, Jensen R, Church S, Wang M, Stiegler G (2005). Anti-human immunodeficiency virus type 1 (HIV-1) antibodies 2F5 and 4E10 require surprisingly few crucial residues in the membrane-proximal external region of glycoprotein gp41 to neutralize HIV-1.. J Virol.

[57] Li M, Gao F, Mascola JR, Stamatatos L, Polonis VR (2005). Human immunodeficiency virus type 1 env clones from acute and early subtype B infections for standardized assessments of vaccine-elicited neutralizing antibodies.. J Virol.

[58] Wei X, Decker JM, Wang S, Hui H, Kappes JC (2003). Antibody neutralization and escape by HIV-1.. Nature.

[59] Long EM, Rainwater SM, Lavreys L, Mandaliya K, Overbaugh J (2002). HIV type 1 variants transmitted to women in Kenya require the CCR5 coreceptor for entry, regardless of the genetic complexity of the infecting virus.. AIDS Res Hum Retroviruses.

[60] Crooks ET, Jiang P, Franti M, Wong S, Zwick MB (2008). Relationship of HIV-1 and SIV envelope glycoprotein trimer occupation and neutralization.. Virology.

[61] Sharma S, Cantwell M, Kipps TJ, Friedmann T (1996). Efficient infection of a human T-cell line and of human primary peripheral blood leukocytes with a pseudotyped retrovirus vector.. Proc Natl Acad Sci U S A.

[62] Buchacher A, Predl R, Strutzenberger K, Steinfellner W, Trkola A (1994). Generation of human monoclonal antibodies against HIV-1 proteins; electrofusion and Epstein-Barr virus transformation for peripheral blood lymphocyte immortalization.. AIDS Res Hum Retroviruses.

[63] Nelson JD, Brunel FM, Jensen R, Crooks ET, Cardoso RM (2007). An Affinity-Enhanced Neutralizing Antibody against the Membrane-Proximal External Region of Human Immunodeficiency Virus Type 1 gp41 Recognizes an Epitope between Those of 2F5 and 4E10.. J Virol.

[64] Cavacini L, Duval M, Song L, Sangster R, Xiang SH (2003). Conformational changes in env oligomer induced by an antibody dependent on the V3 loop base.. Aids.

[65] Peden K, Emerman M, Montagnier L (1991). Changes in growth properties on passage in tissue culture of viruses derived from infectious molecular clones of HIV-1LAI, HIV-1MAL, and HIV-1ELI.. Virology.

[66] Pastore C, Picchio GR, Galimi F, Fish R, Hartley O (2003). Two mechanisms for human immunodeficiency virus type 1 inhibition by N-terminal modifications of RANTES.. Antimicrob Agents Chemother.

[67] Kirschner M, Monrose V, Paluch M, Techodamrongsin N, Rethwilm A (2006). The production of cleaved, trimeric human immunodeficiency virus type 1 (HIV-1) envelope glycoprotein vaccine antigens and infectious pseudoviruses using linear polyethylenimine as a transfection reagent.. Protein Expr Purif.

[68] Poss M, Overbaugh J (1999). Variants from the diverse virus population identified at seroconversion of a clade A human immunodeficiency virus type 1-infected woman have distinct biological properties.. J Virol.

[69] Freed EO, Martin MA (1996). Domains of the human immunodeficiency virus type 1 matrix and gp41 cytoplasmic tail required for envelope incorporation into virions.. J Virol.

[70] Schagger H, Cramer WA, von Jagow G (1994). Analysis of molecular masses and oligomeric states of protein complexes by blue native electrophoresis and isolation of membrane protein complexes by two-dimensional native electrophoresis.. Anal Biochem.

[71] Schulke N, Vesanen MS, Sanders RW, Zhu P, Lu M (2002). Oligomeric and conformational properties of a proteolytically mature, disulfide-stabilized human immunodeficiency virus type 1 gp140 envelope glycoprotein.. J Virol.

[72] Crooks ET, Tong T, Osawa K, Binley JM (2011). Enzyme Digests Eliminate Non-Functional Env from HIV-1 Particle Surfaces Leaving Native Env Trimers Intact and Viral Infectivity Unaffected.. J Virol.

[73] Liao Z, Graham DR, Hildreth JE (2003). Lipid rafts and HIV pathogenesis: virion-associated cholesterol is required for fusion and infection of susceptible cells.. AIDS Res Hum Retroviruses.

[74] Westervelt P, Gendelman HE, Ratner L (1991). Identification of a determinant within the human immunodeficiency virus 1 surface envelope glycoprotein critical for productive infection of primary monocytes.. Proc Natl Acad Sci U S A.

[75] Gendelman HE, Orenstein JM, Martin MA, Ferrua C, Mitra R (1988). Efficient isolation and propagation of human immunodeficiency virus on recombinant colony-stimulating factor 1-treated monocytes.. J Exp Med.

[76] Koyanagi Y, Miles S, Mitsuyasu RT, Merrill JE, Vinters HV (1987). Dual infection of the central nervous system by AIDS viruses with distinct cellular tropisms.. Science.

[77] Salzwedel K, West JT, Hunter E (1999). A conserved tryptophan-rich motif in the membrane-proximal region of the human immunodeficiency virus type 1 gp41 ectodomain is important for Env-mediated fusion and virus infectivity.. J Virol.

[78] Karlsson GB, Gao F, Robinson J, Hahn B, Sodroski J (1996). Increased envelope spike density and stability are not required for the neutralization resistance of primary human immunodeficiency viruses.. J Virol.

[79] Wu X, Zhou T, O'Dell S, Wyatt RT, Kwong PD (2009). Mechanism of human immunodeficiency virus type 1 resistance to monoclonal antibody B12 that effectively targets the site of CD4 attachment.. J Virol.

[80] Binley JM, Cayanan CS, Wiley C, Schulke N, Olson WC (2003). Redox-triggered infection by disulfide-shackled human immunodeficiency virus type 1 pseudovirions.. J Virol.

[81] Kassa A, Finzi A, Pancera M, Courter JR, Smith AB (2009). Identification of a human immunodeficiency virus type 1 envelope glycoprotein variant resistant to cold inactivation.. J Virol.

[82] Alvarez M, Matamoros T, Menendez-Arias L (2009). Increased thermostability and fidelity of DNA synthesis of wild-type and mutant HIV-1 group O reverse transcriptases.. J Mol Biol.

[83] Layne SP, Merges MJ, Spouge JL, Dembo M, Nara PL (1991). Blocking of human immunodeficiency virus infection depends on cell density and viral stock age.. J Virol.

[84] Magnus C, Rusert P, Bonhoeffer S, Trkola A, Regoes RR (2009). Estimating the stoichiometry of human immunodeficiency virus entry.. J Virol.

[85] Layne SP, Merges MJ, Dembo M, Spouge JL, Conley SR (1992). Factors underlying spontaneous inactivation and susceptibility to neutralization of human immunodeficiency virus.. Virology.

[86] Mansy SS, Szostak JW (2008). Thermostability of model protocell membranes.. Proc Natl Acad Sci U S A.

[87] Moore MD, Fu W, Soheilian F, Nagashima K, Ptak RG (2008). Suboptimal inhibition of protease activity in human immunodeficiency virus type 1: effects on virion morphogenesis and RNA maturation.. Virology.

[88] Casali M, Zambonelli C, Goldwasser J, Vu HN, Yarmush ML (2008). Moloney murine leukemia virus decay mediated by retroviral reverse transcriptase degradation of genomic RNA.. Virology.

[89] Kahle KM, Steger HK, Root MJ (2009). Asymmetric deactivation of HIV-1 gp41 following fusion inhibitor binding.. PLoS Pathog.

[90] Amin N, Liu AD, Ramer S, Aehle W, Meijer D (2004). Construction of stabilized proteins by combinatorial consensus mutagenesis.. Protein Eng Des Sel.

[91] Cowan DA (1997). Thermophilic proteins: stability and function in aqueous and organic solvents.. Comp Biochem Physiol A Physiol.

[92] Beringue V, Mallinson G, Kaisar M, Tayebi M, Sattar Z (2003). Regional heterogeneity of cellular prion protein isoforms in the mouse brain.. Brain.

[93] Ruprecht CR, Krarup A, Reynell L, Mann AM, Brandenberg OF (2011). MPER-specific antibodies induce gp120 shedding and irreversibly neutralize HIV-1.. J Exp Med.

[94] Yang X, Mahony E, Holm GH, Kassa A, Sodroski J (2003). Role of the gp120 inner domain beta-sandwich in the interaction between the human immunodeficiency virus envelope glycoprotein subunits.. Virology.

[95] Cao J, Bergeron L, Helseth E, Thali M, Repke H (1993). Effects of amino acid changes in the extracellular domain of the human immunodeficiency virus type 1 gp41 envelope glycoprotein.. J Virol.

[96] York J, Nunberg JH (2004). Role of hydrophobic residues in the central ectodomain of gp41 in maintaining the association between human immunodeficiency virus type 1 envelope glycoprotein subunits gp120 and gp41.. J Virol.

[97] Lorizate M, de la Arada I, Huarte N, Sanchez-Martinez S, de la Torre BG (2006). Structural analysis and assembly of the HIV-1 Gp41 amino-terminal fusion peptide and the pretransmembrane amphipathic-at-interface sequence.. Biochemistry.

[98] Gach JS, Leaman DP, Zwick M (2011). Targeting HIV-1 gp41 in close proximity to the membrane using antibody and other molecules.. Curr Top Med Chem.

[99] Epand RM, Sayer BG, Epand RF (2003). Peptide-induced formation of cholesterol-rich domains.. Biochemistry.

[100] Kassa A, Madani N, Schon A, Haim H, Finzi A (2009). Transitions to and from the CD4-bound conformation are modulated by a single-residue change in the human immunodeficiency virus type 1 gp120 inner domain.. J Virol.

[101] Finzi A, Xiang SH, Pacheco B, Wang L, Haight J (2010). Topological layers in the HIV-1 gp120 inner domain regulate gp41 interaction and CD4-triggered conformational transitions.. Mol Cell.

[102] Beddows S, Zheng NN, Herrera C, Michael E, Barnes K (2005). Neutralization sensitivity of HIV-1 Env-pseudotyped virus clones is determined by co-operativity between mutations which modulate the CD4-binding site and those that affect gp120-gp41 stability.. Virology.

[103] Peters PJ, Duenas-Decamp MJ, Sullivan WM, Brown R, Ankghuambom C (2008). Variation in HIV-1 R5 macrophage-tropism correlates with sensitivity to reagents that block envelope: CD4 interactions but not with sensitivity to other entry inhibitors.. Retrovirology.

[104] Reeves JD, Gallo SA, Ahmad N, Miamidian JL, Harvey PE (2002). Sensitivity of HIV-1 to entry inhibitors correlates with envelope/coreceptor affinity, receptor density, and fusion kinetics.. Proc Natl Acad Sci U S A.

[105] Poignard P, Fouts T, Naniche D, Moore JP, Sattentau QJ (1996). Neutralizing antibodies to human immunodeficiency virus type-1 gp120 induce envelope glycoprotein subunit dissociation.. J Exp Med.

[106] Popovic M, Tenner-Racz K, Pelser C, Stellbrink HJ, van Lunzen J (2005). Persistence of HIV-1 structural proteins and glycoproteins in lymph nodes of patients under highly active antiretroviral therapy.. Proc Natl Acad Sci U S A.

[107] Santosuosso M, Righi E, Lindstrom V, Leblanc PR, Poznansky MC (2009). HIV-1 envelope protein gp120 is present at high concentrations in secondary lymphoid organs of individuals with chronic HIV-1 infection.. J Infect Dis.

[108] Klein JS, Bjorkman PJ (2010). Few and far between: how HIV may be evading antibody avidity.. PLoS Pathog.

[109] Zhu P, Liu J, Bess J, Chertova E, Lifson JD (2006). Distribution and three-dimensional structure of AIDS virus envelope spikes.. Nature.

[110] Or-Guil M, Wittenbrink N, Weiser AA, Schuchhardt J (2007). Recirculation of germinal center B cells: a multilevel selection strategy for antibody maturation.. Immunol Rev.

[111] Igarashi T, Brown C, Azadegan A, Haigwood N, Dimitrov D (1999). Human immunodeficiency virus type 1 neutralizing antibodies accelerate clearance of cell-free virions from blood plasma.. Nat Med.

[112] Huber M, Fischer M, Misselwitz B, Manrique A, Kuster H (2006). Complement lysis activity in autologous plasma is associated with lower viral loads during the acute phase of HIV-1 infection.. PLoS Med.

